# Peptidoglycan Recycling Promotes Outer Membrane Integrity and Carbapenem Tolerance in Acinetobacter baumannii

**DOI:** 10.1128/mbio.01001-22

**Published:** 2022-05-31

**Authors:** Nowrosh Islam, Misha I. Kazi, Katie N. Kang, Jacob Biboy, Joe Gray, Feroz Ahmed, Richard D. Schargel, Cara C. Boutte, Tobias Dörr, Waldemar Vollmer, Joseph M. Boll

**Affiliations:** a Department of Biology, University of Texas—Arlington, Arlington, Texas, USA; b Centre for Bacterial Cell Biology, Biosciences Institute, Newcastle University, Newcastle upon Tyne, United Kingdom; c Biosciences Institute, Newcastle University, Newcastle upon Tyne, United Kingdom; d Weill Institute for Cell and Molecular Biology, Cornell University, Ithaca, New York, USA; e Department of Microbiology, Cornell University, Ithaca, New York, USA; f Cornell Institute of Host-Microbe Interactions and Disease, Cornell University, Ithaca, New York, USA; University of Texas Southwestern Medical Center Dallas

**Keywords:** tolerance, peptidoglycan, outer membrane, cell envelope, carbapenems, Gram-negative bacteria

## Abstract

β-Lactam antibiotics exploit the essentiality of the bacterial cell envelope by perturbing the peptidoglycan layer, typically resulting in rapid lysis and death. Many Gram-negative bacteria do not lyse but instead exhibit “tolerance,” the ability to sustain viability in the presence of bactericidal antibiotics for extended periods. Antibiotic tolerance has been implicated in treatment failure and is a stepping-stone in the acquisition of true resistance, and the molecular factors that promote intrinsic tolerance are not well understood. Acinetobacter baumannii is a critical-threat nosocomial pathogen notorious for its ability to rapidly develop multidrug resistance. Carbapenem β-lactam antibiotics (i.e., meropenem) are first-line prescriptions to treat A. baumannii infections, but treatment failure is increasingly prevalent. Meropenem tolerance in Gram-negative pathogens is characterized by morphologically distinct populations of spheroplasts, but the impact of spheroplast formation is not fully understood. Here, we show that susceptible A. baumannii clinical isolates demonstrate tolerance to high-level meropenem treatment, form spheroplasts upon exposure to the antibiotic, and revert to normal growth after antibiotic removal. Using transcriptomics and genetic screens, we show that several genes associated with outer membrane integrity maintenance and efflux promote tolerance, likely by limiting entry into the periplasm. Genes associated with peptidoglycan homeostasis in the periplasm and cytoplasm also answered our screen, and their disruption compromised cell envelope barrier function. Finally, we defined the enzymatic activity of the tolerance determinants penicillin-binding protein 7 (PBP7) and ElsL (a cytoplasmic ld-carboxypeptidase). These data show that outer membrane integrity and peptidoglycan recycling are tightly linked in their contribution to A. baumannii meropenem tolerance.

## INTRODUCTION

The cell envelope is a dynamic barrier composed of an inner (cytoplasmic) membrane, a periplasm that includes a thin peptidoglycan (PG) layer, and an outer membrane, which is a selective barrier that restricts the entry of toxins and antibiotics. While the PG layer is known to protect against bursting due to cell turgor, the outer membrane also contributes to protection against lysis when external osmotic conditions change (1). Perturbation of the outer membrane or PG envelope layers induces lysis in many bacteria, but regulated responses that fortify the envelope can maintain envelope homeostasis to promote pathogen survival during stress exposure (2).

Antibiotic treatment failure is a growing threat to public health and has primarily been associated with antibiotic resistance (i.e., growth with antibiotic treatment). However, antibiotic tolerance, a population’s ability to survive otherwise toxic levels of antibiotic treatment for extended periods, likely acts as a stepping-stone to true resistance (3–5). Antibiotic tolerance is characterized by the survival of cell populations in a nondividing state, where the MIC does not change, and cells revert to normal growth when the antibiotic is removed, degraded, or diluted (6–8). Molecular factors that extend survival during treatment increase the probability of resistance-conferring mutations or the occurrence of horizontal gene transfer (3).

Carbapenems are important β-lactam therapeutics because they possess potent broad-spectrum activity and are not susceptible to common resistance mechanisms (9, 10). In fact, meropenem is a last-line carbapenem antibiotic used to treat multidrug-resistant Gram-negative infections (11, 12). While meropenem treatment is typically reserved to fight multidrug-resistant bacteria, it is a first-line prescription against the highly drug-resistant nosocomial pathogen Acinetobacter baumannii (13, 14). Carbapenem-resistant A. baumannii has become commonplace among hospital-acquired infections. In 2019, the Centers for Disease Control and Prevention listed carbapenem-resistant A. baumannii as one of the most urgent threats to public health (15), and a report by the World Health Organization prioritized the pathogen as critical for new antibiotic development (16), underscoring its clinical significance.

We reasoned that since tolerance is a prerequisite for true resistance, tolerance factors may be widespread among meropenem-susceptible A. baumannii strains. Defining intrinsic tolerance factors in A. baumannii may offer fundamental insight into how resistance mechanisms rapidly spread among populations and provide new targets to combat tolerant pathogens. While our understanding of resistance mechanisms that cause antibiotic treatment failure has been well documented, tolerance factors that precede the acquisition of true resistance are limited.

Here, we show that susceptible A. baumannii strains, including laboratory-adapted and recent clinical isolates, survive for extended periods (>24 h) in high levels of meropenem, demonstrating widespread tolerance. Meropenem induces cell wall-deficient spheroplast formation in A. baumannii, as shown in other Gram-negative pathogens (17–19). After the removal of the antibiotic, cells readily revert to the canonical A. baumannii coccobacillus morphology and resume growth. Time-resolved transcriptome sequencing during meropenem treatment showed the differential expression of genes that coordinate a regulatory response to reduce the intracellular meropenem concentration. In addition, we found that PG recycling was also a major contributor to A. baumannii survival during meropenem treatment, and the disruption of genes encoding periplasmic and cytoplasmic PG maintenance enzymes compromises outer membrane integrity. Finally, we also define the enzymatic activities of two novel tolerance determinants, penicillin-binding protein 7 (PBP7) (encoded by *pbpG*) and ElsL (also known as LdtK [20]). Together, these studies show that A. baumannii coordinates several pathways to limit meropenem-induced cell envelope damage. These findings provide new targets to direct antimicrobial therapies that optimize the effective treatment of A. baumannii and prevent the spread of resistance.

## RESULTS

### Meropenem-susceptible A. baumannii strains are tolerant, form spheroplasts, and resume normal morphology and growth upon removal of the bactericidal antibiotic.

Previous work showed that Vibrio cholerae (17, 18), Pseudomonas aeruginosa (19), and pathogens of the *Enterobacterales* order (21, 22) form viable, nondividing spheroplasts when exposed to lethal concentrations of β-lactam antibiotics over several hours. Importantly, spheroplasts revert to normal rod-shaped growth when the antibiotic concentration is sufficiently reduced (21), demonstrating a short-term survival mechanism that directly contributes to antibiotic treatment failure.

**FIG 1:**
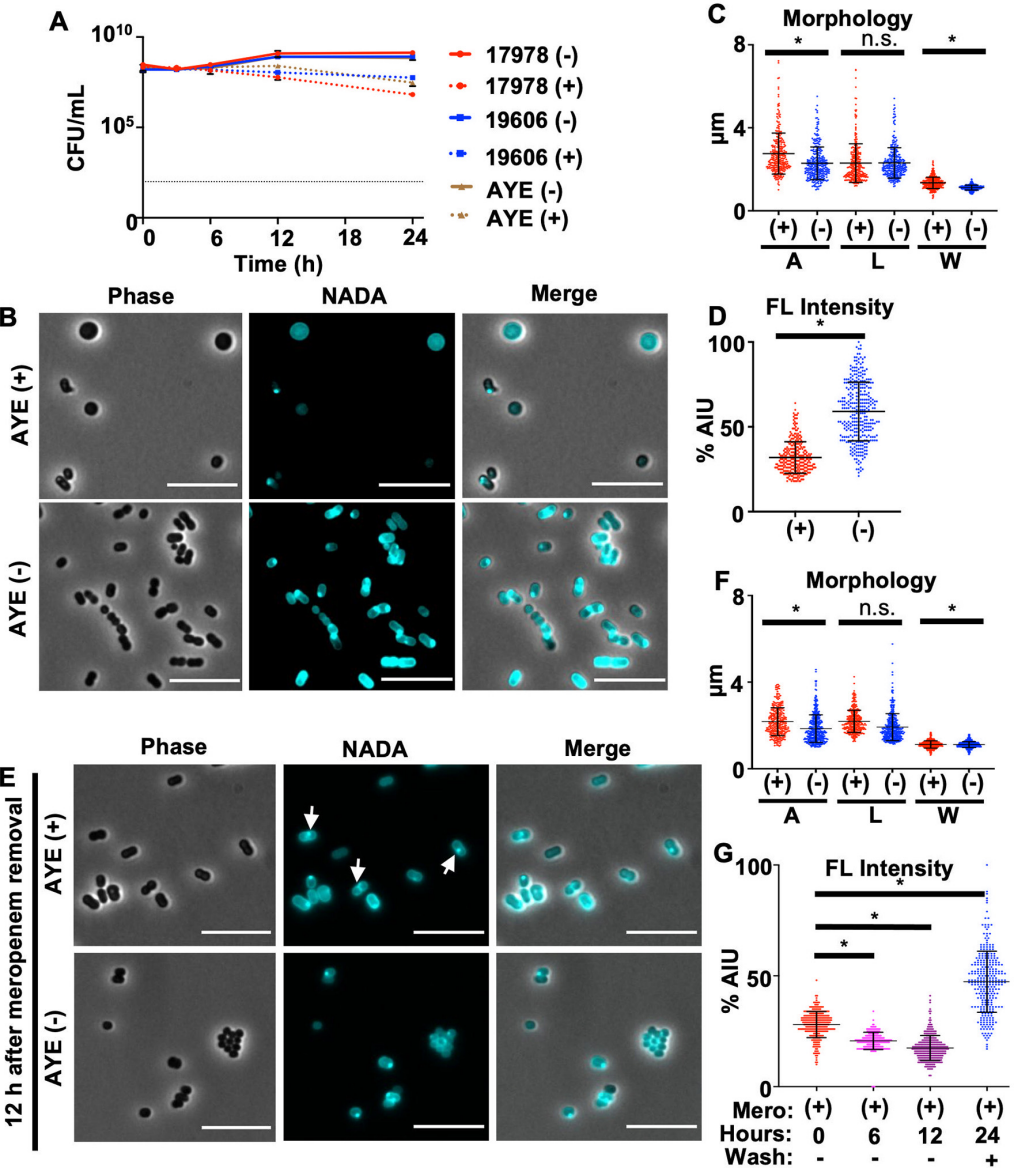
Acinetobacter baumannii strains are tolerant to meropenem. (A) CFU of A. baumannii strains ATCC 17978, ATCC 19606, and AYE untreated (−) or treated (+) with meropenem over 24 h. Each killing assay was independently replicated three times, and one representative data set is reported. Error bars indicate standard deviations (SD) from the means. The dotted black line indicates the level of detection. (B) Phase-contrast and fluorescence microscopy of treated or untreated A. baumannii strain AYE after 12 h. Bars, 10 μm. (C) Area (A), length (L), and width (W) quantitations of cells in panel B (*n* = 300). (D) Fluorescence (FL) signal intensity quantitation in percent arbitrary intensity units (AIU) of treated versus untreated cells in panel B (*n* = 300). (E) After 12 h of meropenem treatment, the antibiotic was removed, and cells were resuspended in fresh medium without the antibiotic and stained with NADA. Cells were imaged after a 12-h recovery period and showed that the characteristic coccobacillus morphology was restored. White arrows indicate fluorescence intensity at the midcell. (F) Area, length, and width (W) of cells in panel E (*n* = 300). (G) Fluorescence signal intensity in percent AIU for treated versus untreated cells (*n* = 300) at 0, 6, and 12 h during meropenem treatment and 12 h after the removal of the antibiotic. Significance was determined using an unpaired *t* test (*P* < 0.05) in treated versus untreated cells. An asterisk indicates significant differences between treated and untreated cells. n.s., not significant. Error bars indicate SD from the means.

To determine if populations of A. baumannii strains can tolerate meropenem treatment over time, stationary-phase cultures from susceptible A. baumannii isolates, including recent clinical isolates, were treated with high levels (10 μg/mL; 62.5-fold the MIC for strain ATCC 17978) of the antibiotic. Treated cultures demonstrated only a slight decrease in viability after 24 h, relative to untreated cultures (Fig. 1A; see also Fig. S1A in the supplemental material). In contrast, meropenem treatment of cells in the logarithmic growth phase caused rapid lysis (Fig. S1B). Therefore, A. baumannii strains are highly tolerant to normally lethal meropenem concentrations during slow growth, a relevant physiological state during infection when the cell is known to fortify the cell envelope (23). While these data agree with the current dogma that β-lactam-dependent killing is strictly proportional to the growth rate (7, 24, 25), subsequent analysis revealed that stationary-phase A. baumannii cells experience significant cell envelope damage upon meropenem treatment. After 12 h, stationary-phase cells treated with meropenem demonstrated notable morphological changes relative to untreated cells (Fig. 1B; Fig. S2). All strains showed measurable increases in the surface area and width of treated cells relative to untreated cells (Fig. 1C; Fig. S2). To visualize changes in PG assembly, cells were treated with a fluorescent derivative of d-alanine [NBD-(linezolid-7-nitrobenz-2-oxa-1,3-diazol-4-yl)-amino-d-alanine (NADA)], which is incorporated into the peptidoglycan by PBPs and ld-transpeptidases (26–29). A significant decrease in the NADA intensity was evident in meropenem-treated cultures relative to untreated cultures at 12 h (Fig. 1B and D; Fig. S2), suggesting the degradation of the cell wall and spheroplast formation, as previously shown in other β-lactam-tolerant Gram-negative bacteria (17, 21). Thus, tolerance in stationary phase is not just a simple function of growth inhibition but rather is an active response to significant cell envelope damage in A. baumannii.

10.1128/mbio.01001-22.1 FIG S1 Tolerance in clinical A. baumannii isolates. (A) Dilution spot assays of A. baumannii strain ATCC 17978 and four recent clinical isolates, including a resistant (CDC273), an intermediate (CDC293), and two meropenem-susceptible (CDC280 and CDC300) strains for comparison. The calculated meropenem MIC is indicated below each image. (B) Survival (CFU per milliliter) of A. baumannii strains ATCC 17978 and AYE in logarithmic-phase cultures was calculated over 12 h during meropenem treatment. Each experiment was independently replicated two times, and one representative data set is reported. The dotted black line indicates the level of detection. Error bars represent the averages from 3 technical replicates ± SD from the means. Download FIG S1, TIF file, 0.9 MB. Copyright © 2022 Islam et al. 2022 Islam et al. https://creativecommons.org/licenses/by/4.0/ This content is distributed under the terms of the Creative Commons Attribution 4.0 International license.

10.1128/mbio.01001-22.2 FIG S2 Spheroplast formation in A. baumannii isolates after 12 h of meropenem treatment. Shown are phase-contrast and fluorescence microscopy images of treated (+) and untreated (−) A. baumannii strains after 12 h. Bar, 10 μm. To the right of each image are fluorescence (FL) intensity quantifications reported in percent arbitrary intensity units (AIU) in treated (red) versus untreated (blue) cells and cell shape quantifications, including area (A), length (L), and width (W) (*n *= 300). Significance was determined using an unpaired *t* test (*P* < 0.05) in treated versus untreated cells. Error bars indicate SD relative to the means. An asterisk indicates significant differences (*P* < 0.05). n.s., not significant. Download FIG S2, TIF file, 1.7 MB. Copyright © 2022 Islam et al. 2022 Islam et al. https://creativecommons.org/licenses/by/4.0/ This content is distributed under the terms of the Creative Commons Attribution 4.0 International license.

Since meropenem-treated A. baumannii spheroplasts were viable after plating (Fig. 1A; Fig. S1A), we also wanted to determine if the characteristic A. baumannii coccobacillus morphology was restored after antibiotic removal. Cells treated with meropenem for 12 h were washed and grown in fresh medium without the antibiotic. At 12 h posttreatment, no spheroplasts were found after antibiotic removal (Fig. 1E), and wild-type (WT) morphology was largely restored (Fig. 1F), suggesting that PG was being synthesized and remodeled (Fig. 1E). We also tracked the cell wall content during meropenem exposure and subsequent recovery. Fluorescence intensity measurements showed a stepwise decrease in the fluorescence intensity 6 and 12 h after treatment relative to the start of the experiment (0 h) (Fig. 1G), confirming PG degradation during meropenem treatment. After 12 h of meropenem treatment, cells were washed to remove the antibiotic, resuspended in fresh medium, and again stained with NADA. Fluorescence intensity measurements 12 h after meropenem treatment showed a drastic increase in NADA incorporation (Fig. 1G), suggesting that the cell resumed PG remodeling and synthesis. Furthermore, the fluorescence intensity appeared higher at the midcell of some cells (Fig. 1E, white arrows), suggesting that the recovered population had resumed division. Together, these data support a model where A. baumannii spheroplasts revert to wild-type morphology and growth when meropenem treatment is stopped.

### Transcriptome analysis highlights differentially regulated pathways with a putative role in A. baumannii tolerance.

**FIG 2:**
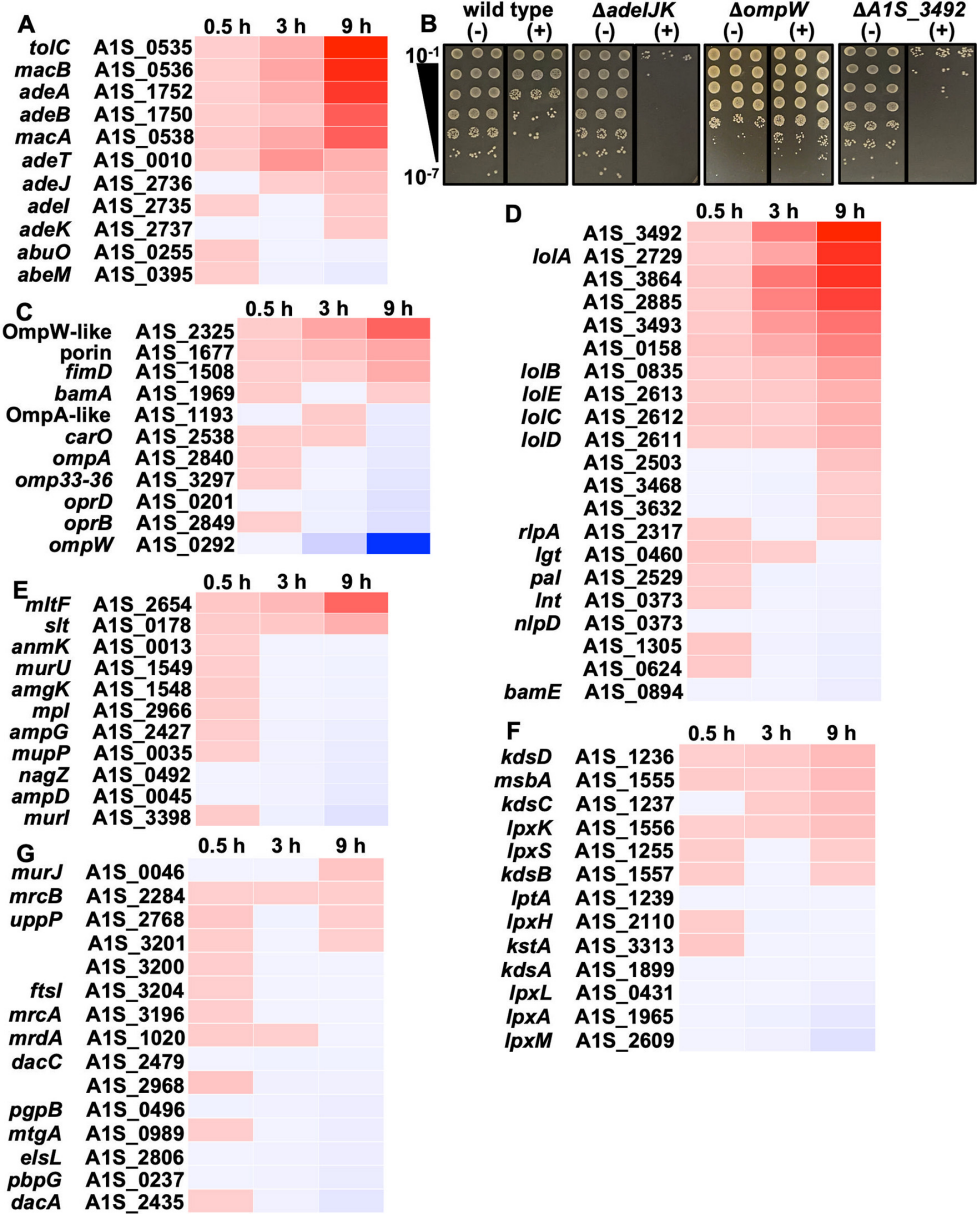
Differentially regulated genes in response to meropenem treatment in A. baumannii. Heat maps show the fold changes in genes expressed at 0.5, 3, and 9 h of meropenem treatment relative to wild-type ATCC 17978 (*P* < 0.05). (A) Differentially regulated genes associated with efflux. (B) Dilution spot assays (in triplicate) of wild-type, Δ*adeIJK*, Δ*ompW*, and Δ*A1S_3492* strains with (+) or without (−) meropenem treatment. (C to G) Same as panel A but with pathway analysis including genes associated with outer membrane porins (C), outer membrane lipoproteins and their transporters (D), PG recycling (E), LOS biosynthesis (F), and PG biosynthesis (G).

Many Gram-negative pathogens rapidly form tolerant spheroplasts during meropenem exposure (17, 21); however, A. baumannii spheroplast formation is delayed. We first observed spheroplast formation only after 8 h, with large numbers within the population accumulating by 12 h (Fig. 1B and C; Fig. S2). To define transcriptional alterations associated with spheroplast-associated tolerance, we isolated RNA from treated and untreated cells at 0.5, 3, and 9 h. While subtle changes in gene expression were evident at 0.5 and 3 h, differential expression patterns were more obvious at 9 h in treated cultures relative to untreated cultures (Fig. 2). Genes associated with efflux were increasingly upregulated with each time point (Fig. 2A), suggesting that the cell quickly and continually responds to meropenem treatment by actively expelling the toxic compound. Upregulated efflux genes included *adeAB*, *adeIJK*, and *macAB-tolC*, all of which have been implicated in antibiotic efflux (30–32); specifically, β-lactam efflux is associated with the AdeIJK RND-type pump (33, 34). To validate the role of AdeIJK in meropenem tolerance or resistance, we constructed a genetic knockout (Δ*adeIJK*), which was subjected to high-level meropenem treatment (Fig. 2B). At 24 h posttreatment, the Δ*adeIJK* mutant was more than 1,000-fold more susceptible to meropenem-mediated killing than the wild type, showing that efflux contributes to tolerance.

Porins represent the major entryway for carbapenems such as meropenem to enter the periplasm (35), where they inhibit transpeptidation to cross-link the stem peptides of adjacent PG strands. Decreased expression of many porin-associated genes was evident in treated cultures relative to untreated cultures (Fig. 2C), suggesting that the cell also limits meropenem entry by reducing porin gene expression in response to treatment. However, the temporal expression of porin-associated genes was delayed relative to efflux, in general. For example, the porin genes *carO* and *oprD* were downregulated at later time points only (Fig. 2C). The deletion of *carO* is associated with carbapenem resistance in A. baumannii (36), and it was found to be an influx channel for carbapenems (37), while OprD has also been associated with clinical carbapenem resistance in A. baumannii (38), suggesting that reduced expression may strategically limit meropenem entry. Interestingly, the largest reduction in gene expression was associated with *ompW*, which encodes a predicted β-barrel protein (OmpW) that supports iron uptake (39), but our understanding of its biological function or how it contributes to carbapenem resistance or tolerance is limited. Notably, in Vibrio cholerae, decreased iron uptake regulated by the VxrAB two-component system promotes spheroplast recovery by reducing oxidative stress during β-lactam treatment (40, 41), potentially suggesting a similar iron reduction role for the downregulation of *ompW*. To further validate the RNA sequencing data set, we analyzed the Δ*ompW* mutant in a meropenem killing assay, which showed an ~10-fold increase in survival relative to the wild type (Fig. 2B). Thus, the downregulation of *ompW* contributes to tolerance.

Consistent with previously reported A. baumannii transcriptional data sets under stress conditions (42–44), meropenem treatment also induces the expression of genes encoding putative outer membrane lipoproteins and their transporters (LolA to -D) (Fig. 2D). Outer membrane lipoproteins fortify the Escherichia coli cell envelope by providing structural rigidity, where some outer membrane lipoproteins are covalently attached to the underlying PG network (1, 45). Finally, we made an isogenic mutant of *A1S_3492*, encoding a putative outer membrane lipoprotein that was strongly upregulated following meropenem exposure. The Δ*A1S_3492* mutant showed an ~100-fold increase in meropenem susceptibility relative to the wild type (Fig. 2B). Together, the analyses of efflux, porin, and lipoprotein mutants suggest a transcriptional response that protects A. baumannii from meropenem-dependent killing during treatment.

The transcription of genes associated with PG remodeling was only slightly altered, with the notable exception of two genes encoding putative lytic transglycosylases, including the outer membrane-bound protein MltF and a soluble protein, Slt, both of which were upregulated (Fig. 2E). Lytic transglycosylases were recently shown to fulfill an important role in clearing un-cross-linked or mis-cross-linked periplasmic muropeptide fragments, which can accumulate to detrimental levels in the presence of β-lactams, potentially explaining the adaptive value of their upregulation (46, 47). Finally, genes involved in lipooligosaccharide (LOS) and PG biosynthesis were slightly altered (Fig. 2F and G).

### Genes and pathways that contribute to A. baumannii fitness during meropenem treatment.

**FIG 3:**
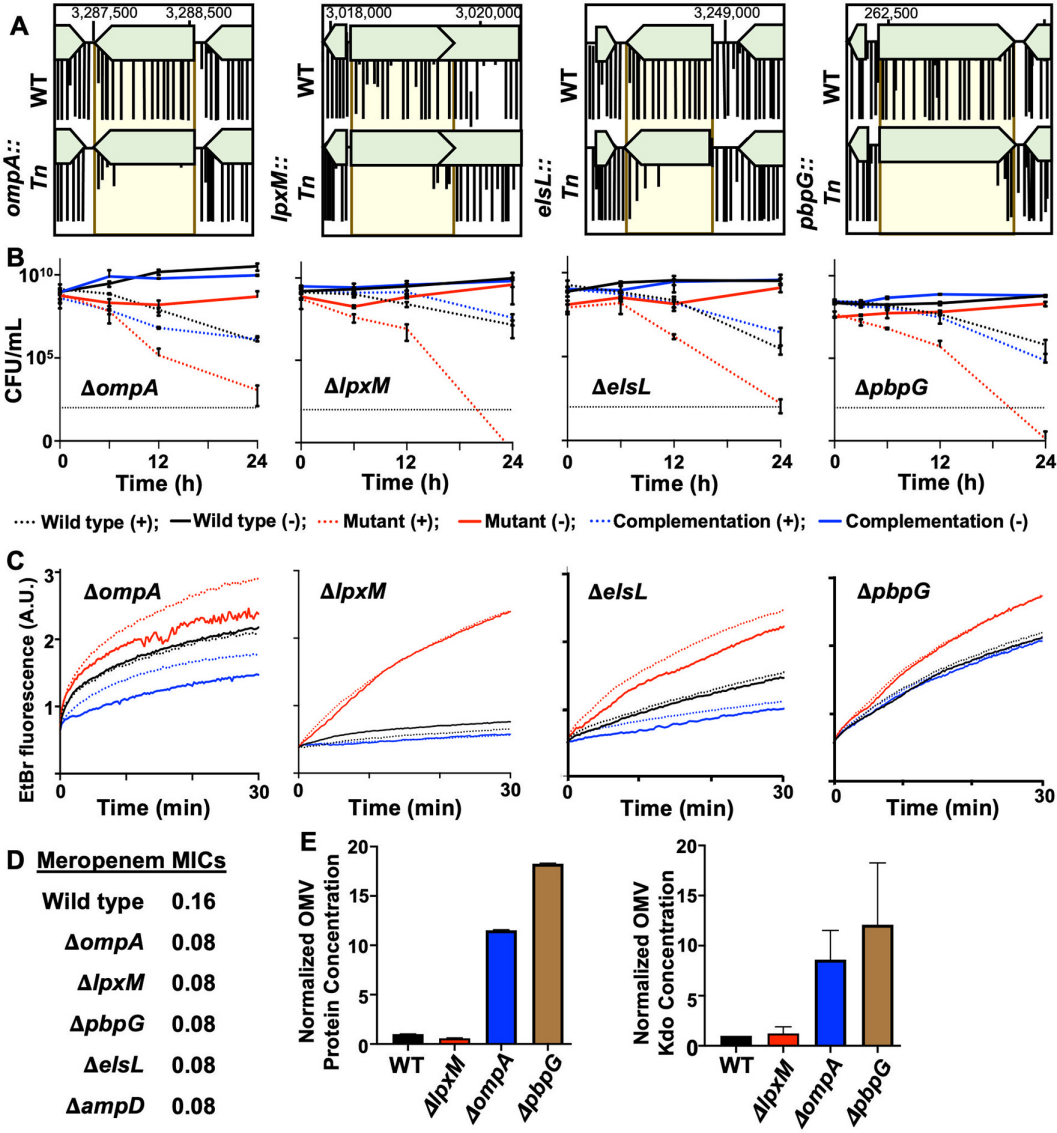
Genes encoding outer membrane integrity and PG maintenance contribute to meropenem tolerance in A. baumannii. (A) Tn-seq analysis of genes required for meropenem tolerance at 12 h. (B) Survival in isogenic mutants was calculated as CFU per milliliter over 24 h during meropenem treatment. Data were collected from two experiments in triplicate. Error bars represent the averages from 3 technical replicates ± SD. (C) Permeability assays using ethidium bromide (EtBr) over 0.5 h. A.U., arbitrary units. Lines depict the means from three technical replicates. (D) MICs of wild-type and isogenic A. baumannii mutant strains. (E) Relative quantification of protein (left) and Kdo (right) concentrations of outer membrane vesicles (OMVs) in wild-type (WT) and mutant strains. Each experiment was independently replicated three times, and one representative data set is reported. Error bars indicate SD from the means. An asterisk indicates significant differences relative to the WT strain (*P* < 0.05).

While transcriptome sequencing analyses offer insight into specific stress responses, one limitation is that differentially regulated genes often do not impact fitness due to redundancy or pleiotropic effects. Therefore, we also performed transposon sequencing (Tn-seq) on A. baumannii strain ATCC 17978. Using previously constructed high-density transposon libraries generated in wild-type A. baumannii (20, 48), stationary-phase cultures were either treated with meropenem or left untreated during incubation at 37°C. After 6, 9, and 12 h, cells were collected, insertions were mapped, and comparisons between treated and untreated cultures were used to identify meropenem tolerance factors. The screen was answered by several novel factors, some of which are the subject of a separate study, but also revealed the importance of outer membrane integrity and PG maintenance (Fig. 3A). To validate our screen, we assessed survival in the presence and absence of meropenem in several mutants, including Δ*ompA*, Δ*lpxM*, Δ*pbpG*, and Δ*elsL* (also known as *ldtK* [20]) (Fig. 3B). All mutants showed at least a 2- to 3-log fold reduction in viable counts relative to the wild type at 12 h and a >5-log fold reduction at 24 h. Importantly, the meropenem MICs did not change significantly in the mutants relative to the wild type (Fig. 3D). These studies suggest that A. baumannii fitness during meropenem treatment is dependent on outer membrane integrity and PG maintenance factors.

OmpA is a highly conserved monomeric β-barrel protein with a periplasmic domain that noncovalently attaches the outer membrane to the PG network (49). It is highly abundant in A. baumannii (50) and coordinates with efflux pumps to export antibacterial compounds from the periplasm (51, 52). OmpA is also known to stabilize the outer membrane; *ompA* deletion/disruption induces outer membrane vesicle formation and increases permeability (53). To test the hypothesis that *ompA* deletion perturbs the outer membrane to promote meropenem entry in A. baumannii, we performed two assays, including permeability measurements (Fig. 3C) and outer membrane vesiculation assays (Fig. 3E). Relative to the wild type, the Δ*ompA* mutant exhibited increased outer membrane vesicle formation and permeability to ethidium bromide (EtBr), which is similar in size to meropenem. We also measured ethidium bromide influx in the Δ*lpxM*, Δ*pbpG*, and Δ*elsL* mutants (Fig. 3C). Like the Δ*ompA* mutant, all isogenic mutants had increased permeability relative to the wild type and the respective complementation strains, which restored the permeability defect. Permeability was notably reduced beyond the wild type in Δ*ompA* and Δ*elsL* complementation strains, which was likely due to protein overexpression (Fig. S3A and B). Furthermore, meropenem treatment did not exacerbate permeability in the wild type or any of the mutants (Fig. 3C), suggesting that it does not directly destabilize outer membrane barrier function. Since we previously reported that the Δ*elsL* mutant produces excess outer membrane vesicles (20), and all of the mutants showed increased permeability, we also tested vesicle formation in the Δ*lpxM* and Δ*pbpG* mutants (Fig. 3E). Unexpectedly, the Δ*pbpG* mutant produced excess outer membrane vesicles relative to the wild type and all other mutants. In contrast, the Δ*lpxM* mutant did not.

10.1128/mbio.01001-22.3 FIG S3 Analysis of mutant A. baumannii strains. (A and B) Whole-cell lysates from wild-type, mutant, and complementation strains were subjected to immunoblot analysis using anti-OmpA (38.44 kDa) (A) and anti-ElsL (18.97 kDa) (B) polyclonal antisera. Anti-RpoA (37.26 kDa) was used as a loading control. (C to F) Phase-contrast and fluorescence microscopy images of NADA-treated wild-type and Δ*ompA* (C), Δ*lpxM* (D), Δ*pbpG* (E), and Δ*elsL* (F) strains with the respective complementation strains. Bar, 10 μm. Fluorescence (FL) signal intensity quantification in percent arbitrary intensity units (AIU) in wild-type, mutant, and complementation strain are shown to the right of the images. Cell shape quantifications, including area (A), length (L), and width (W) (*n* = 300), are also included. Significance was determined using an unpaired *t* test (*P* < 0.05). An asterisk indicates significant differences between the wild type and the mutant (*P* < 0.05). n.s., not significant. Error bars indicate SD from the means. Download FIG S3, TIF file, 2.1 MB. Copyright © 2022 Islam et al. 2022 Islam et al. https://creativecommons.org/licenses/by/4.0/ This content is distributed under the terms of the Creative Commons Attribution 4.0 International license.

Interestingly, the Δ*lpxM* mutant was the only strain that showed increased permeability but not hypervesiculation. LpxM catalyzes the transfer of two lauroyl (C_12:0_) groups from an acyl carrier protein to the *R*-3′- and *R*-2-hydroxymyristate positions of lipid A during LOS biosynthesis (54). Mutations that reduce LOS acylation are known to increase the fluidity of the lipid bilayer and could also impact the folding/function of outer membrane porins (55, 56). Either or both of these mechanisms could increase the entry of meropenem into the periplasmic space or disrupt efflux mechanisms that actively pump the compound out of the cell.

We also characterized the morphology of each mutant during growth (Fig. S3C to F). We found that relative to the wild type, Δ*ompA* cells were chained, and NADA incorporation was reduced (Fig. S3C), suggesting that OmpA is required for the proper function of PG enzymes (division proteins and ld/dd-transpeptidases that incorporate NADA and/or increase carboxypeptidase activity). Δ*lpxM* and Δ*pbpG* mutants showed increased NADA incorporation (Fig. S3D and E), which is consistent with increased outer membrane permeability (or decreased carboxypeptidase activity). Δ*pbpG* cells were also clumped (Fig. S3E), suggesting that the cells could not properly separate during division. As previously reported (20), the Δ*elsL* mutant exhibited cell rounding (Fig. S3F).

### PBP7 is a dd-carboxypeptidase and -endopeptidase that catalyzes the formation of tetrapeptides.

**FIG 4:**
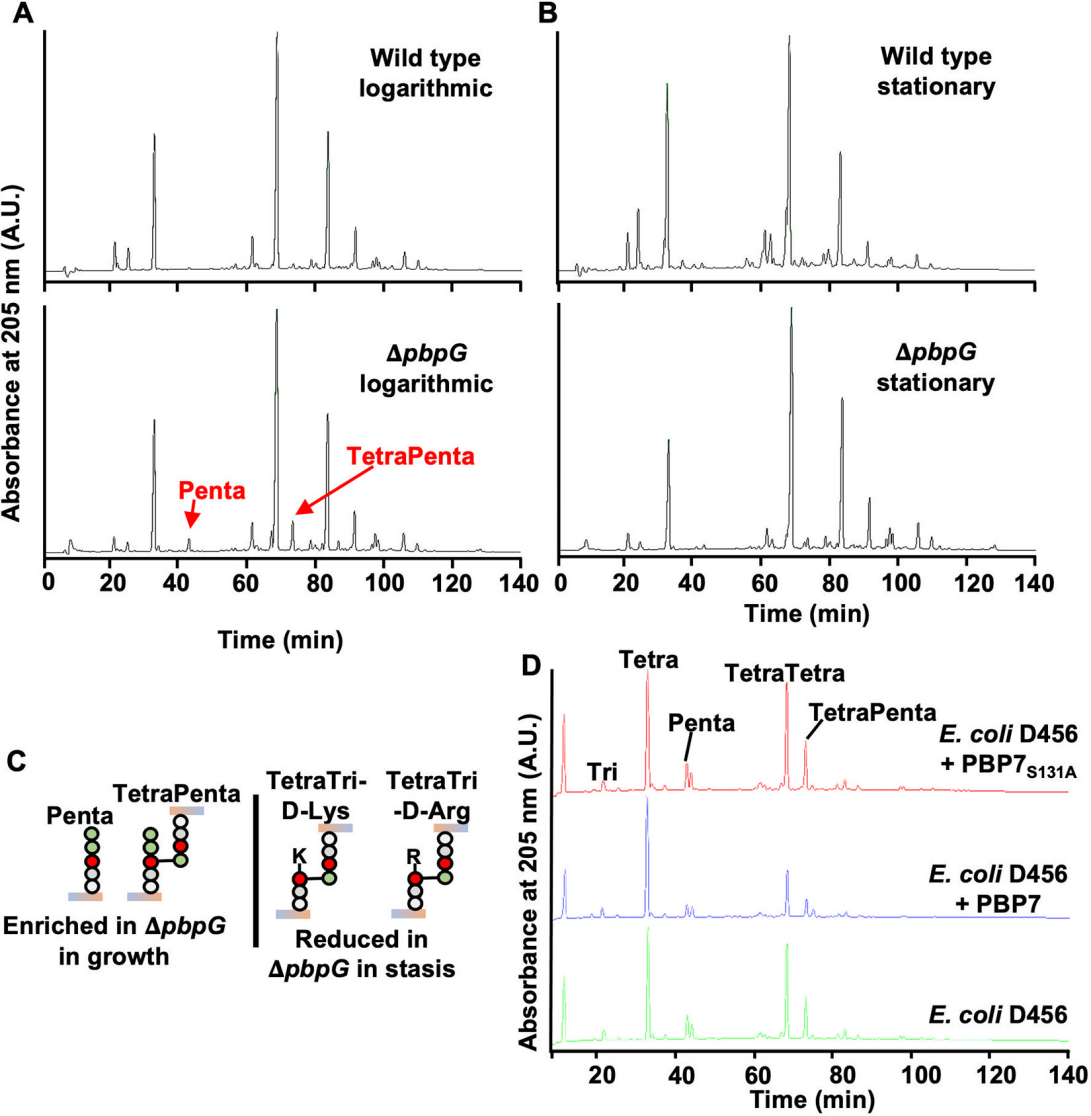
PBP7 is active against pentapeptides and dd-cross-links. (A) PG isolated from wild-type and Δ*pbpG* strains in the growth phase was analyzed by HPLC. The muropeptides Penta and TetraPenta were enriched in the Δ*pbpG* mutant. (B) PG isolated from wild-type and Δ*pbpG* strains in stationary phase was analyzed by HPLC. TetraTri-d-Lys and TetraTri-d-Arg were depleted in the Δ*pbpG* mutant relative to the wild type. (C) Muropeptide structures are illustrated and were confirmed using MS/MS. (D) Recombinant PBP7 or the active-site mutant PBP7_S131A_ was incubated with PG isolated from E. coli D456, which contains Tetra, Penta, TetraTetra, and TetraPenta as the main muropeptides. PBP7 was active against pentapeptides (dd-carboxypeptidase) and cross-linked muropeptides (dd-edopeptidase).

We were intrigued by the genetic links of tolerance to PG maintenance (i.e., *pbpG* and *elsL*). Mutation of *pbpG* and *elsL* impacted outer membrane integrity (Fig. 3), but we sought to more precisely define their physiological role to determine specific pathways that contribute to meropenem tolerance. To define the activity of A. baumannii PBP7 (encoded by *pbpG*), *in vivo*, we isolated PG from exponential- and stationary-phase cultures of wild-type and Δ*pbpG* strains (Fig. 4A and B; Table S1). Muropeptides were generated by treatment with muramidase and separated by high-performance liquid chromatography (HPLC), and uncharacterized peaks were analyzed by tandem mass spectrometry (MS/MS) (Fig. S4), as done previously (20, 42, 57). The PG composition of the Δ*pbpG* mutant during exponential growth displayed an accumulation of two muropeptide peaks that were not present in the wild type (Fig. 4A; Table S1). MS analysis identified these peaks as disaccharide pentapeptide (Penta) (neutral mass, 1,012.19 atomic mass units [amu]; theoretical mass, 1,012.45 amu) and bis-disaccharide tetrapentapeptide (TetraPenta) (neutral mass, 1,935.60 amu; theoretical mass, 1,935.84 amu) (Fig. S4). This PG architecture suggests that these enriched muropeptide pools represent PBP7 substrates during growth. Δ*pbpG* PG during stasis exhibited a reduction in d-amino-acid-modified muropeptide pools, including TetraTri-d-Lys and TetraTri-d-Arg, and reduced 3-3 cross-link formation (Fig. 4B and C; Table S1). This is consistent with PBP7 exhibiting dd-carboxypeptidase activity, providing the abundant tetrapeptide substrate (20, 42, 57) required for ld-transpeptidases. The periplasmic ld-transpeptidase LdtJ transfers d-amino acid to tetrapeptides and forms 3-3 cross-links (20). Therefore, it is likely that PBP7 provides at least some of the periplasmic substrates for LdtJ-dependent transpeptidase activity during stasis.

10.1128/mbio.01001-22.4 FIG S4 Unidentified peaks in the logarithmic growth phase of the Δ*pbpG* mutant. Peaks at 42 min (A) and 72 min (B) were analyzed by mass spectrometry (MS). The peak in panel A was consistent with disaccharide pentapeptide (Penta) (neutral mass, 1,012.19 amu; theoretical mass, 1,012.45 amu), and the peak in panel B was consistent with bis-disaccharide tetrapentapeptide (TetraPenta) (neutral mass, 1,935.60 amu; theoretical mass, 1,935.84 amu). Download FIG S4, TIF file, 0.5 MB. Copyright © 2022 Islam et al. 2022 Islam et al. https://creativecommons.org/licenses/by/4.0/ This content is distributed under the terms of the Creative Commons Attribution 4.0 International license.

10.1128/mbio.01001-22.6 TABLE S1 Muropeptide composition of wild-type and Δ*pbpG* A. baumannii strain ATCC 17978. Download Table S1, DOCX file, 0.02 MB. Copyright © 2022 Islam et al. 2022 Islam et al. https://creativecommons.org/licenses/by/4.0/ This content is distributed under the terms of the Creative Commons Attribution 4.0 International license.

We next tested the *in vitro* enzymatic activity. We purified recombinant PBP7 (Fig. S5A) and a predicted catalytically inactive version in which alanine replaces the active-site serine (PBP7_S131A_). Purified proteins were incubated with PG from the E. coli strain D456 (Fig. 4D), a strain enriched in pentapeptide abundance (58), and analyzed as previously described (59). PBP7 was active against penta-, tetratetra-, and tetrapentapeptides, where each muropeptide was trimmed to the tetrapeptide form relative to the no-enzyme control. In addition, PBP7 efficiently cleaved tetrapeptide dimers into their monomers, demonstrating dd-endopeptidase activity. As expected, PBP7_S131A_ did not show activity against any muropeptides. Together, these studies suggest that PBP7 has both carboxypeptidase and endopeptidase activities and that both activities enrich the periplasmic pool of monomeric tetrapeptides.

10.1128/mbio.01001-22.5 FIG S5 Purification of recombinant PBP7 and ElsL. (A) PBP7_His8×_. (Left) Coomassie-stained SDS-PAGE gel with 1, 2, or 10 μg of recombinant PBP7_His8×_ or PBP7_S131A His8×_ loaded. (Right) Western blotting of 1 μg PBP7_His8×_ or PBP7_S131A His8×_ using an anti-His antibody. (B) ElsL_His8×_. (Left) Coomassie-stained SDS-PAGE gel with 10 μg of recombinant ElsL_His8×_ or ElsL_S131A His8×_ loaded. (Right) Western blotting with 1 μg of each recombinant protein using an anti-penta-His antibody. Download FIG S5, TIF file, 0.4 MB. Copyright © 2022 Islam et al. 2022 Islam et al. https://creativecommons.org/licenses/by/4.0/ This content is distributed under the terms of the Creative Commons Attribution 4.0 International license.

### ElsL is a cytoplasmic ld-carboxypeptidase active against tetrapeptides for PG recycling.

During β-lactam treatment, autolysins (i.e., lytic transglycosylases) are activated (7, 60), which increases the amount of PG turnover products with 1,6-anhydro-*N*-Acetylmuramic acid (Mur*N*Ac) residues. In A. baumannii, genes encoding the autolysins MltF and Slt were upregulated during meropenem treatment (Fig. 2E), which likely increases the periplasmic concentrations of TetraAnh for cytoplasmic import. In E. coli, TetraAnh is the substrate for the ld-carboxypeptidase LdcA, which trims tetrapeptides to tripeptides (61) that are catabolized by the conserved muramidase NagZ (62, 63) and the amidase AmpD (64, 65) to generate 1,6-anhydro-Mur*N*Ac-tripeptide and free tripeptides; the latter can be further broken down into individual amino acids and used as nutrients. Furthermore, Mpl (66) can attach tripeptides to UDP-Mur*N*Ac to form UDP-Mur*N*Ac-tripeptide, an intermediate in the *de novo* PG biosynthesis pathway. However, no apparent ld-carboxypeptidase orthologue of LdcA is encoded by A. baumannii.

**FIG 5:**
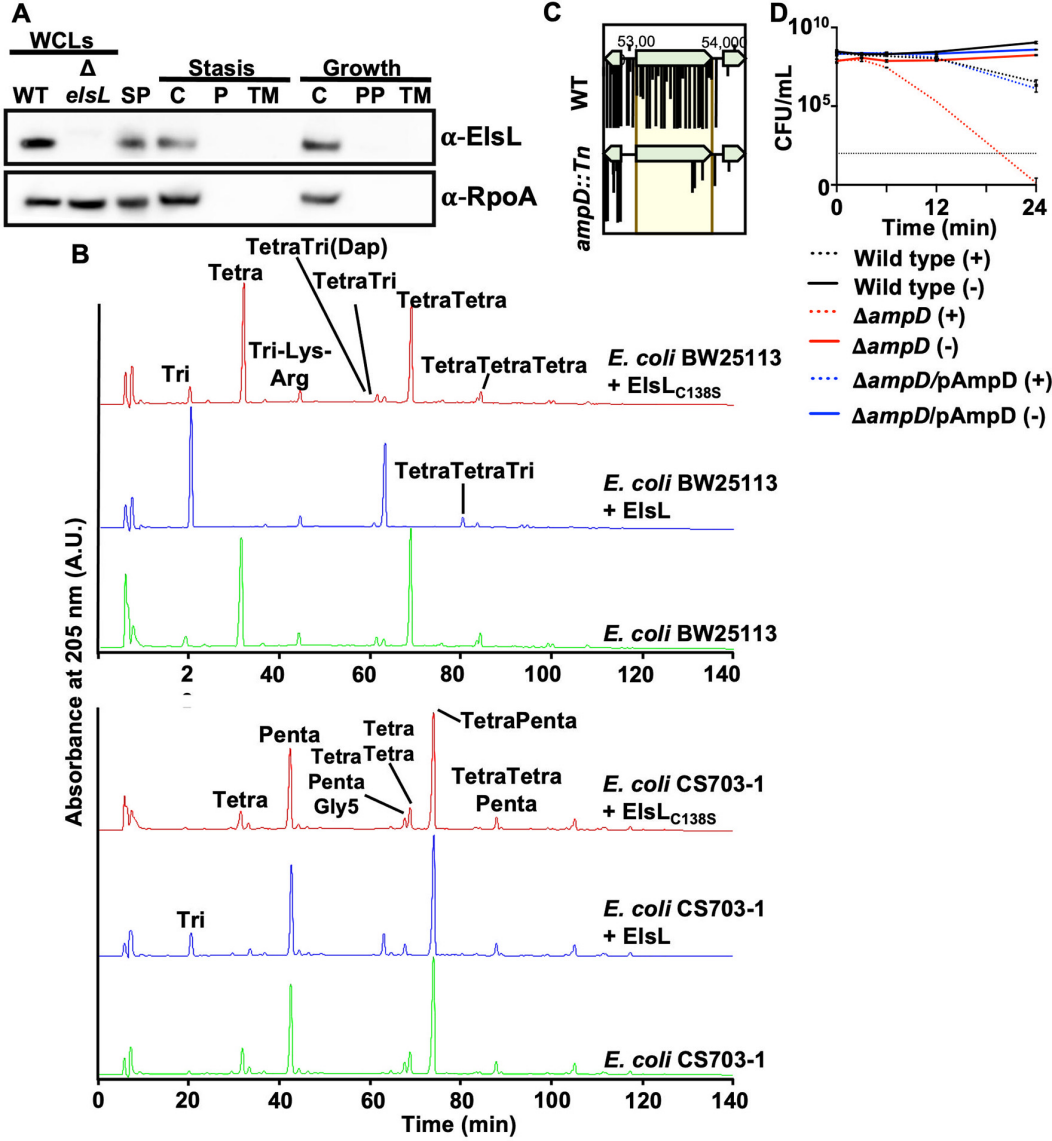
ElsL is active against tetrapeptides in Tetra and TetraTetra. (A) Western blot analysis with anti-ElsL and anti-RpoA antisera. ElsL is 18.97 kDa, while RpoA is 37.27 kDa. WCLs, whole-cell lysates; SP, spheroplast; C, cytoplasm; PP, periplasm; TM, total membrane fraction. (B) Recombinant ElsL or the active-site mutant ElsL_C13S_ was incubated with PG isolated from E. coli strain BW25113 (tetrapeptide rich) (top) or strain CS703-1 (pentapeptide rich) (bottom). ElsL was active against tetrapeptides but not pentapeptides. (C) Tn-seq analysis of *ampD*::Tn insertions relative to the wild type 12 h after meropenem treatment. (D) Meropenem tolerance assay in the Δ*ampD* mutant, which encodes a well-conserved cytoplasmic enzyme required for PG recycling. Error bars represent the averages from 3 technical replicates ± SD from the means.

While this study was in progress, two other groups reported a characterization of ElsL as a cytoplasmic ld-carboxypeptidase (67, 68). Our results here support this conclusion. We confirmed the cytoplasmic localization of ElsL with a specific antibody that detects the native protein (Fig. 5A). After fractionation of the subcellular compartments, we were able to detect ElsL only in the cytoplasmic fraction during growth and stasis, showing that it is not exported to the periplasm.

Since ElsL is cytoplasmic, we sought to determine if it was active on tetra- and/or pentapeptide substrates. We purified recombinant ElsL and the active-site mutant ElsL_C138S_ (Fig. S5B). Both enzymes were incubated with muropeptides obtained from tetrapeptide-rich PG from E. coli BW25113 or pentapeptide-rich PG from E. coli CS703-1 (59) (Fig. 5B). ElsL showed activity against tetrapeptides but not pentapeptides. The muropeptide profile revealed the formation of disaccharide tripeptide and bis-disaccharide tetratripeptide, demonstrating that ElsL cleaves the bond between the l-center of *meso*-diaminopimelic acid (*m*DAP) and the terminal d-Ala in tetrapeptides, confirming ld-carboxypeptidase activity as part of the cytoplasmic PG recycling pathway.

To further confirm that the cytoplasmic PG pathway contributes to meropenem tolerance in A. baumannii, we made an isogenic mutant of *ampD*, which encodes *N*-acetylmuramyl-l-alanine amidase that releases the tripeptide from anhMur*N*Ac (65). Importantly, *ampD* transposon insertions were also depleted in the meropenem tolerance screen (Fig. 5C). Like the Δ*pbpG* and Δ*elsL* mutants, the Δ*ampD* mutant was also rapidly killed when treated with meropenem relative to the wild type and the respective complementation strains (Fig. 5D). Together, these studies strongly suggest that the PG recycling pathway contributes to meropenem tolerance in A. baumannii. Furthermore, the formation of cytoplasmic tripeptides or tetrapeptides appears to contribute to meropenem tolerance. Combinatorial therapies that inhibit enzymes in both PG biosynthesis and recycling could provide an alternative treatment strategy.

## DISCUSSION

Many susceptible Gram-negative pathogens tolerate treatment with bactericidal antibiotics such as carbapenem β-lactams, but the molecular factors that underlie cell survival are not understood. Here, we show that meropenem treatment induces spheroplast formation in A. baumannii during treatment in stationary phase and that cell growth resumes upon the removal of the antibiotic. Transcriptome sequencing analysis suggested that A. baumannii responds to meropenem treatment by fortifying the structural integrity of the cell envelope through increased outer membrane lipoprotein and transporter gene expression. Intriguingly, autolysins were also induced, which likely contribute to tolerance via the removal of detrimental cell wall breakdown products or through their role in initiating PG recycling. Alternatively, autolysins might contribute to cell integrity by producing the necessary precursors for the formation of β-lactam-resistant 3-3 cross-links. Meropenem-treated cells also appear to limit high periplasmic accumulation of the antibiotic through the induced expression of efflux-associated genes and the downregulation of porin genes, both of which reduce periplasmic meropenem concentrations by actively pumping the antibiotic out of the cell and limiting entry, respectively.

**FIG 6:**
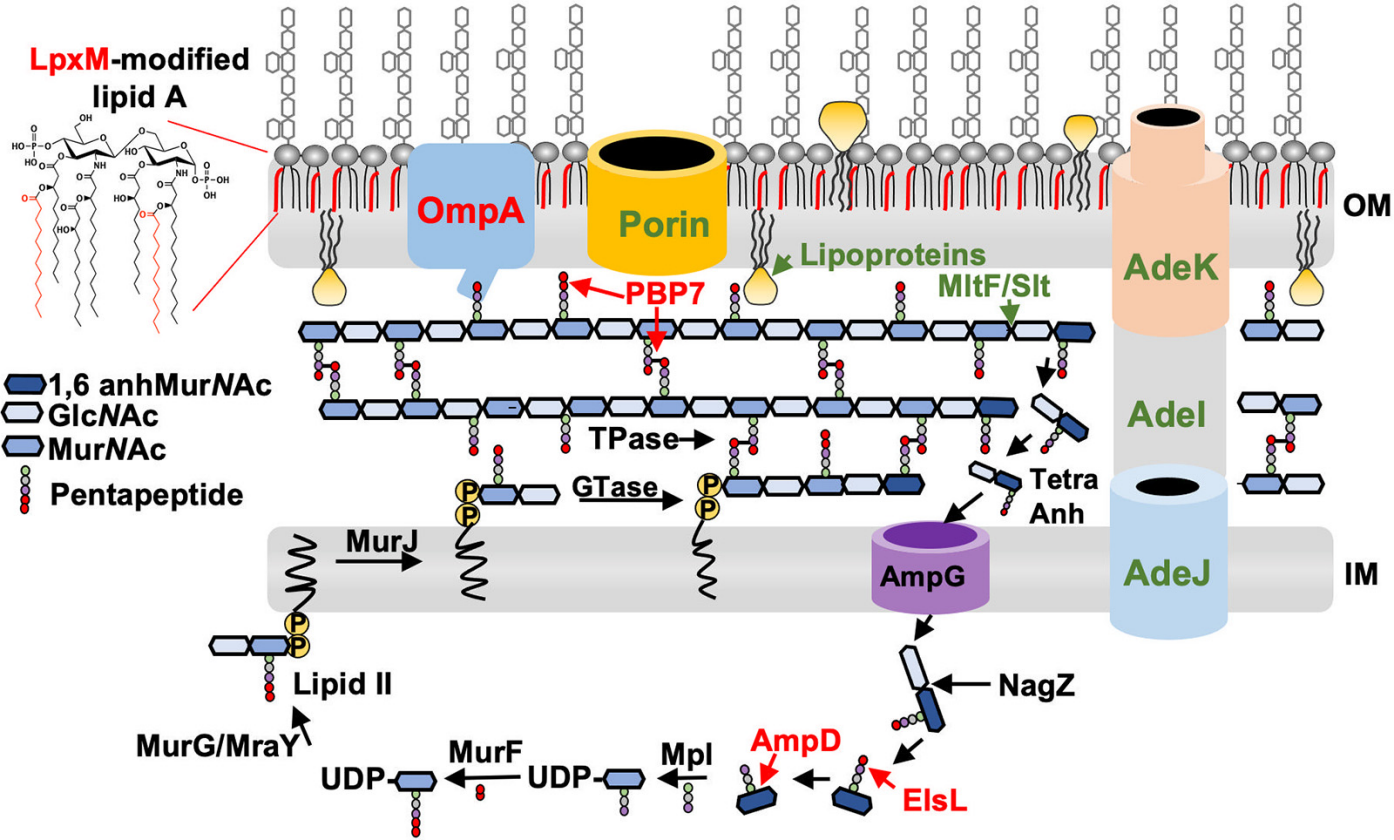
Model showing mechanisms that promote meropenem tolerance in A. baumannii. Based on transcriptomic analysis, several genes are differentially expressed after treatment with high levels of meropenem. Pathways include the upregulation of the AdeIJK efflux pump, lipoproteins, and the MltF/Slt autolysins, while porins, including CarO and OprD, were downregulated (green). Fitness screens showed that several genes involved in the outer membrane (OM), periplasmic, and cytoplasmic pathways promote meropenem tolerance. Pathways include OmpA, which tethers the outer membrane to the peptidoglycan; LpxM, which increases hydrophobic packing in the outer membrane; the dd-carboxypeptidase and dd-endopeptidase PBP7; and the cytoplasmic recycling enzymes ElsL and AmpD (red). Autolysin activity is shown at the 1,6-anhMur*N*Ac end of the glycan chain, but it is not known from which ends MltF/Slt release the muropeptide. It is also possible that ElsL could act before NagZ. IM, inner membrane; Tpases, transpeptidase; GTase, glycosyltransferase.

A separate genetic (transposon) screen to identify fitness determinants revealed factors required for high-level meropenem tolerance and included genes that contribute to outer membrane permeability (*lpxM*, *ompA*, *pbpG*, and *elsL*) and cell envelope stability (*ompA*, *pbpG*, and *elsL*). Furthermore, genes in the cytoplasmic PG recycling pathway, *elsL* and *ampD*, also answered the screen. Together, the data from transcriptomics and genetic screens suggested that factors working to maintain cell envelope homeostasis through integrity maintenance of the outer membrane and PG network contribute to meropenem tolerance in A. baumannii (Fig. 6).

While we showed that several tolerance factors are transcriptionally regulated, we do not know which transcriptional regulators control these responses. A previous study found that PhoPQ-dependent outer membrane modifications promoted survival in cell wall-deficient spheroplasts (22), presumably by fortifying the outer membrane to counter large loads of turgor pressure typically absorbed by the cell wall. Specifically, PhoPQ was activated in response to meropenem treatment. A. baumannii does not encode PhoPQ, but analogous mechanisms are likely to contribute to cell envelope homeostasis to counter the turgor when the cell wall is compromised. One mechanism might include fortification of the cell envelope with lipoproteins, which occurs in A. baumannii during stress (42–44, 69); however, the underlying protective mechanism is not understood. Furthermore, noncovalent attachments between the outer membrane and PG network via OmpA and the hyperacylation of lipid A via LpxM may also increase the mechanical load-bearing capacity of the outer membrane to maintain envelope homeostasis when the cell wall is defective. It is also possible that disruption of OmpA or LpxM induced pleiotropic effects that reduced the barrier function to gate meropenem entry.

Unexpectedly, our data suggest that PG maintenance enzymes contribute to A. baumannii survival during meropenem treatment. Tetrapeptides represent the most abundant PG stem peptides in A. baumannii. They are formed, in part, by the dd-carboxypeptidase and dd-endopeptidase activity of PBP7 on pentapeptides and dd-cross-linked muropeptides, respectively (Fig. 4A). Tetrapeptides are substrates for ld-transpeptidase that form a small amount of 3-3 cross-links in A. baumannii (20) but are needed to effectively repair PG defects in stressed E. coli cells (2). Furthermore, tetrapeptides are also necessary for the ld-transpeptidase-dependent covalent attachment of Braun’s lipoprotein (Lpp) to *m*DAP residues in PG (70), which also fortifies the E. coli envelope (1). Our data indicate that without PBP7, LdtJ-dependent 3-3 cross-link formation is reduced (Fig. 4A), suggesting that PBP7’s role in cell envelope stability is related to its involvement in the 3-3 cross-link formation pathway. However, <3% of all muropeptides contain 3-3 cross-links, and their contributions to PG integrity maintenance remain unclear.

MltF and Slt were induced during meropenem treatment (Fig. 2 and 6), consistent with the activation of autolysins in response to penicillin-binding protein inhibition during β-lactam treatment (7, 17). Lytic transglycosylases cleave the glycosidic linkage between disaccharide subunits within the PG strands and perform intramolecular transglycosylation in Mur*N*Ac to release soluble 1,6-anhydroMur*N*Ac-containing muropeptides, which can be imported into the cytoplasm. The main turnover product, 1,6-anhydro-MurNAc-tetrapeptide (TetraAnh), is transported into the cytoplasm by AmpG, where they provide the substrates for ElsL-dependent ld-carboxypeptidase activity to form TriAnh. Like other members of the YkuD family, ElsL retains a preference for tetrapeptide substrates (Fig. 5B) but represents the first known YkuD-containing enzyme that lacks a signal sequence and is active in the cytoplasm. ElsL is the second member of the YkuD family, after DpaA/LdtF, that has a major role in cleaving amide bonds rather than generating them. Notably, the requirement for ElsL in meropenem tolerance implies that cells depend on PG maintenance during spheroplast formation. Finally, ElsL (and LdtJ) was shown to be essential for A. baumannii survival without LOS (20), suggesting that PG recycling and modification of tetrapeptides are a general response to counter cell envelope stress in A. baumannii. It is also possible that the cytosolic accumulation of tetrapeptides somehow exacerbates mutant sickness.

The hydrolysis of TriAnh by the dedicated enzymes NagZ and AmpD results in the formation of anhMur*N*Ac-tripeptide and of 1,6-anhMur*N*Ac and tripeptides, respectively, and these fragments could be further degraded into individual amino acids for utilization as nutrient or energy sources (71–73) to promote survival during tolerance. It is reasonable to expect that the cell requires some nutrients during tolerance, and this pathway could provide energy to support basal metabolic processes. Alternatively, Mpl could ligate tripeptides to UDP-Mur*N*Ac in the recycling pathway (66, 74). UPD-Mur*N*Ac-tripeptide is an intermediate in the *de novo* PG synthesis pathway (75–77), and it is possible that the enhanced availability of PG precursors primes cells for efficient recovery when the antibiotic has been removed. Another possibility is that the accumulation of cytoplasmic 1,6-anhydroMur*N*Ac-containing muropeptides provides signals to induce β-lactamase expression, which could degrade meropenem. Notably, two mechanisms have been characterized in Gram-negative bacteria, including the AmpG-AmpR pathway and the BlrAB two-component system, both of which induce β-lactamase expression in response to muropeptide concentrations (78). While A. baumannii strain ATCC 17978 encodes both Acinetobacter-derived cephalosporinase (encoded by *A1S_2367*) and a putative OXA-51-like β-lactamase (encoded by *A1S_1517*), neither gene was upregulated in the transcriptomics analysis. It is also possible that one or both β-lactamases have low-level carbapenemase activity, which, when coupled with the observed downregulation of porins, might contribute sufficiently to meropenem degradation to promote survival. While many genes in A. baumannii have not yet been characterized, it is also possible that signaling pathways and potentially other carbapenemases could be induced in response to 1,6-anhydroMur*N*Ac-containing muropeptide accumulation to promote meropenem degradation. A more detailed analysis is needed to characterize the PG recycling tolerance mechanism, which will inform more effective treatment strategies to combat A. baumannii infections.

## MATERIALS AND METHODS

### Bacterial strains and growth.

All strains and plasmids used in this study are listed in Table S2 in the supplemental material. Primers are listed in Table S3. All A. baumannii strains were grown aerobically from freezer stocks on Luria-Bertani (LB) agar at 37°C. Antibiotics were used at the following concentrations unless noted otherwise: 25 mg/L kanamycin, 10 mg/L meropenem, 10 mg/L tetracycline, and 75 mg/L carbenicillin.

10.1128/mbio.01001-22.7 TABLE S2 Strains and plasmids used in this study. Download Table S2, DOCX file, 0.03 MB. Copyright © 2022 Islam et al. 2022 Islam et al. https://creativecommons.org/licenses/by/4.0/ This content is distributed under the terms of the Creative Commons Attribution 4.0 International license.

10.1128/mbio.01001-22.8 TABLE S3 Oligonucleotides used in this study. Download Table S3, DOCX file, 0.02 MB. Copyright © 2022 Islam et al. 2022 Islam et al. https://creativecommons.org/licenses/by/4.0/ This content is distributed under the terms of the Creative Commons Attribution 4.0 International license.

### Construction of genetic mutants.

A. baumannii *pbpG*, *ampD*, *ompA*, and *adeIJK* mutants were constructed as described previously (20, 54), using the recombination-mediated genetic engineering (recombineering) method (54). Briefly, a kanamycin resistance cassette flanked by FLP recombination target (FRT) sites was PCR amplified from the pKD4 plasmid using primers containing 125-bp flanking regions of homology to the gene of interest. The resulting linear PCR product was then transformed via electroporation into A. baumannii strain ATCC 17978 expressing pREC*_Ab_* (pAT03). Transformants were recovered in Luria broth and plated onto LB agar supplemented with 7.5 mg/L kanamycin. All genetic mutants were confirmed by PCR.

Following the isolation of genetic mutants, the pMMB67EH::REC*_Ab_* Tet^r^ plasmid was removed as described previously (54). Isolated mutants were grown on LB agar supplemented with 2 mM nickel(II) chloride (NiCl_2_) and replica plated onto LB agar supplemented with kanamycin or tetracycline. The loss of the pMMB67EH::REC*_Ab_* Tet^r^ plasmid in mutants susceptible to tetracycline and resistant to kanamycin was confirmed using PCR. To excise the chromosomal insertion of the kanamycin resistance cassette, cured mutants were transformed with pMMB67EH carrying the FLP recombinase (pAT08) and plated onto LB agar supplemented with tetracycline and 2 mM isopropyl β-d-1-thiogalactopyranoside (IPTG) to induce the expression of FLP recombinase. The successful excision of the kanamycin resistance cassette was confirmed using PCR.

The PBP7 complementation vector was constructed by amplifying the *pbpG* (*A1S_0237*) coding sequence (encoding PBP7) with 200-bp upstream and downstream flanking regions from A. baumannii ATCC 17978 genomic DNA (gDNA) and cloning it into the XhoI and KpnI restriction sites in the pABBRkn^R^ plasmid. The resulting pPBP7 plasmid was transformed into the A. baumannii ATCC 17978 Δ*pbpG* background for complementation using the native promoter.

AmpD and OmpA complementation vectors were constructed similarly, with slight alterations. The *ampD* (*A1S_0045*) and *ompA* (*A1S_2840*) coding sequences were amplified from A. baumannii ATCC 17978 gDNA and cloned into the BamHI and SalI restriction sites in the pMMB67EHkn^R^ plasmid. The resulting pAmpD and pOmpA plasmids were transformed into the respective mutants and induced with 2 mM IPTG for complementation.

### Fluorescent NADA staining.

Cultures were grown overnight with shaking at 37°C in 5 mL of BHI (Bacto brain heart infusion) broth (BD Difco). The following day, cultures were back-diluted at 1:10 in fresh BHI medium (total volume of 5 mL) without or with meropenem. Two microliters of 10 mM NBD-(linezolid-7-nitrobenz-2-oxa-1,3-diazol-4-yl)-amino-d-alanine (NADA) (Thermo Fisher) was added to each tube, and the mixture was incubated at 37°C. At 0, 6, and 12 h, cultures (5 mL) were washed twice in BHI broth and fixed with phosphate-buffered saline (PBS) containing a 1:10 solution of 16% paraformaldehyde. For spheroplast recovery, 12-h-treated cultures were washed 3 times in BHI broth to remove the excess meropenem and resuspended in fresh BHI broth. A total of 10 mM NADA was added, and the mixture was incubated for 12 h at 37°C before fixing the cells for microscopy.

### Microscopy.

Paraformaldehyde-fixed cells were immobilized on 1.5% agarose pads and imaged using an inverted Nikon Eclipse Ti-2 wide-field epifluorescence microscope equipped with a Photometrics Prime 95B camera and a Plan Apo 100× 1.45-numerical-aperture lens objective. Phase-contrast and fluorescence images were collected with NIS Elements software. Green fluorescence images were taken using a Sola light-emitting diode (LED) light engine and a filter cube with 632/60- or 535/50-nm emission filters.

### Image analysis.

Microscopy images were processed and pseudocolored with ImageJ Fiji (79). A cyan lookup table was applied to NADA images. Cell shape (length, width, and fluorescence intensities) and total cell surface area were quantified in MicrobeJ (80), and data were plotted in Prism 9 (GraphPad 9.2.0). Each experiment was independently replicated three times, data from one representative experiment are reported for quantification, and one representative image is included in the figures.

### RNA sequencing.

Transcriptome sequencing analysis was performed as described previously, with modification (42). Briefly, the Direct-Zol RNA miniprep kit (Zymo Research) was used to extract total RNA from A. baumannii ATCC 17978 cultures treated with either meropenem or an equivalent volume of water as a blank at 0.5, 3, and 9 h at 37°C in triplicate. A Turbo DNA-free DNA removal kit (Invitrogen) was used to remove genomic DNA contamination. DNase-depleted RNA was sent to the Microbial Genome Sequencing Center (MiGS) for Illumina NextSeq 550 sequencing. CLC genomic workbench software (Qiagen) was used to align the resulting sequencing data to the A. baumannii ATCC 17978 genome annotations and determine the reads per kilobase per million (RPKM) expression values and the weighted-proportions fold changes in expression values between meropenem-treated and untreated samples. Baggerley’s test on proportions was used to generate a false discovery rate-adjusted *P* value. The weighted-proportions fold change in expression values between samples was used to generate pathway-specific heat maps in Prism 9.

### Transposon insertion sequencing.

Transposon sequencing was performed as described previously (20, 42, 48, 81). Briefly, pJNW684 was conjugated into wild-type A. baumannii strain ATCC 17978 to generate a library of ~400,000 mutants. The transposon mutant library was pooled and screened for survival with and without meropenem treatment at 6, 9, and 12 h at 37°C. gDNA from meropenem-treated and untreated cultures was isolated and sheared, and transposon junctions were amplified and sequenced. The frequencies of transposon insertions were compared between meropenem-treated and untreated conditions to determine fitness determinants that contribute to carbapenem tolerance in A. baumannii.

### Time-dependent killing assays.

Meropenem killing experiments were performed as previously described, with slight alterations (21). Wild-type, mutant, and complementation strains were grown overnight in Luria broth at 37°C. The following day, cultures grown overnight were back-diluted at 1:10 in fresh, prewarmed BHI broth containing meropenem or an equivalent volume of water. Diluted BHI broth cultures were then incubated at 37°C. At 0, 3, 6, 12, and 24 h, each sample was diluted 4-fold in blank BHI broth, and the optical density at 600 nm (OD_600_) was measured. At each time point, cells were serially diluted 10-fold in fresh BHI broth, and either 5 μL of each serial dilution was spot plated or 100 μL of each dilution was plated onto LB agar. Spot plates were imaged and CFU were calculated after 24 h at 37°C. Each experiment was independently replicated three times, and one representative data set is reported.

### Construction of PBP7 and ElsL active-site mutants.

Site-directed mutagenesis was performed, as previously described, with *elsL* (*A1S_2806*) (20). Briefly, the *pbpG* coding sequence was amplified from A. baumannii ATCC 17978 gDNA, cloned into the BamHI restriction site in pUC19, and transformed into E. coli C2987 chemically competent cells (New England BioLabs, Inc.). pUC19::*pbpG* was used as a template for *Pfu*-mediated deletion mutagenesis. DpnI-digested PCR mixtures were transformed into E. coli C2987 chemically competent cells and plated onto LB agar supplemented with 75 mg/L carbenicillin. All mutants were confirmed by PCR and Sanger sequencing.

### Construction of PBP7 and ElsL overexpression strains.

*pbpG* and *elsL* coding sequences were amplified from A. baumannii ATCC 17978 gDNA, and *pbpG*_S131A_ and *elsL*_C138S_ were amplified from pUC19::*pbpG*_S131A_ and pUC19::*elsL*_C138S_ plasmid DNA using primers containing a His8× tag sequence. Amplicons were cloned into the NdeI and BamHI restriction sites in pT7-7Kn and transformed into E. coli C2987 chemically competent cells, resulting in pT7-7Kn::*pbpG*, pT7-7Kn::*pbpG*_S131A_, pT7-7Kn::*elsL*, and pT7-7Kn::*elsL*_C138S_. Constructs were confirmed using Sanger sequencing and transformed into chemically competent E. coli C2527(BL21) cells (New England BioLabs, Inc.) for purification, expression, and Western blotting.

### Purification of recombinant PBP7 and ElsL.

BL21 cells carrying pT7-7Kn::*pbpG*, pT7-7Kn::*pbpG*_S131A_, pT7-7Kn::*elsL*, and pT7-7Kn::*elsL*_C138S_ were grown in 500 mL Luria broth with 1 mM IPTG at 37°C for 7 h. Cells were collected, washed in cold 1× PBS, and pelleted, and the supernatant was removed. The dry pellet was frozen at −80°C overnight. The pellet was thawed on ice and resuspended in 20 mL lysis buffer (20 mM Tris, 300 mM NaCl, 10 mM imidazole [pH 8]). Samples were sonicated for 20 s on and off for 10 min at 60% amplitude (Qsonica Q125 sonicator). Cells were centrifuged at 20,000 × *g* for 0.5 h at 4°C. The supernatant was incubated with lysis buffer-washed HisPur Ni-nitrilotriacetic acid (NTA) resin (Thermo Scientific) on a rotator for 2 h at 4°C. The sample was added to a 10-mL protein purification column containing a porous polyethylene disk (Thermo Scientific) and allowed to gravity drip. The column was washed three times with 20 mL lysis buffer and increasing concentrations of additional imidazole at each wash (0 mM, 15 mM, and 30 mM). Five hundred microliters of elution buffer (20 mM Tris, 300 mM NaCl, 250 mM imidazole [pH 8]) was incubated with the column for 5 min and then gravity eluted 9 times. The elution fractions containing protein, as determined by a protein gel, were injected into a 10-mW dialysis cassette (Thermo Scientific) and dialyzed overnight in dialysis buffer (10 mM Tris, 50 mM KCl, 0.1 mM EDTA, 5% glycerol [pH 8]) at 4°C. Purified protein was collected and verified by Western blotting with an anti-His antibody.

### Isolation of outer membrane vesicles.

Outer membrane vesicles were isolated as described previously (20). Briefly, cultures grown overnight were back-diluted to an OD_600_ of 0.01 in 100 mL Luria broth and grown to stationary phase at 37°C. Cultures were then pelleted at 5,000 × *g* for 15 min at room temperature, and the supernatant was filtered through a 0.45-mm bottle-top filter. The filtered supernatant was ultracentrifuged (Sorvall WX 80+ ultracentrifuge with an AH-629 swinging-bucket rotor) at 151,243 × *g* for 1 h at 4°C. Following a final ultracentrifugation step, the outer membrane vesicle pellet was resuspended in 500 mL cold membrane vesicle buffer (50 mM Tris, 5 mM NaCl, 1 mM MgSO_4_ [pH 7.5]). The isolation of outer membrane vesicles was repeated three times in duplicate; one representative data set is reported.

### Quantification of total outer membrane vesicle proteins.

A Bradford assay was used to determine outer membrane vesicle protein concentrations, as previously described (20). To generate a standard curve, bovine serum albumin (BSA) was diluted at 0 to 20 mg/mL in Pierce Coomassie Plus assay reagent (Thermo Fisher) to a final volume of 1 mL. Outer membrane vesicles were diluted at 2, 5, 10, 15, and 20 μL in reagent to a final volume of 1 mL. A microplate spectrophotometer (Fisherbrand AccuSkan) was used to measure the absorbance (OD_595_) of the standard and samples in a 96-well plate (BrandTech). Protein concentrations were determined by comparing the optical densities of the samples to the standard curve plotted in Microsoft Excel, and final quantifications were graphed in GraphPad Prism 9. Experiments were reproduced three times from each outer membrane vesicle isolation, and one representative data set is reported.

### Quantification of outer membrane vesicle Kdo concentrations.

3-Deoxy-d-manno-oct-2-ulosonic acid (Kdo) assays were carried out as described previously (20, 82). For the standard curve, the Kdo standard (Sigma) was diluted at 0 to 128 μg/mL in 50 μL of deionized (DI) water. Fifty microliters of 0.5 M sulfuric acid (H_2_SO_4_) was added to 50 μL of isolated outer membrane vesicles and freshly prepared 50-μL dilutions of the Kdo standard. Outer membrane versicles in 0.5 M H_2_SO_4_ were boiled for 8 min to release the Kdo sugars. Samples were allowed to cool for 10 min at room temperature. Fifty microliters of 0.1 M periodic acid was added to outer membrane vesicles and Kdo standards, and the mixture was incubated at room temperature for 10 min. Following incubation, 200 μL of 0.2 M sodium arsenite in 0.5 M hydrochloric acid (HCl) was added to outer membrane vesicles and Kdo standards, followed by 800 μL of 0.6% freshly prepared thiobarbituric acid (TBA). All samples were boiled for 10 min and allowed to cool at room temperate for 30 to 40 min. Prior to optical density measurements, purified Kdo was extracted using *n*-butanol equilibrated with 0.5 M HCl. The optical density was measured at 552 nm and 509 nm (Fisherbrand AccuSkan microplate spectrophotometer) in disposable polystyrene cuvettes (Fisherbrand). A linear Kdo standard curve was generated by subtracting OD_552_ measurements from OD_509_ measurements in Microsoft Excel, and final quantifications were graphed in GraphPad Prism 9. Experiments were reproduced three times from each outer membrane vesicle isolation, and one representative data set is reported.

### Ethidium bromide permeability assay.

Permeability assays were done as previously described (83), with slight modifications. Briefly, cultures were grown overnight in 5 mL BHI medium, normalized, and back-diluted (1:10) in BHI medium with and without meropenem. Cultures were withdrawn at 0, 6, and 12 h; washed 3 times with PBS; and normalized based on the OD_600_. One hundred eighty milliliters of the cultures was added to a 96-well black plate, and 6 μM EtBr was added immediately before fluorescence measurements. The relative fluorescence units (RFU) were analyzed using a synergy multimode plate reader (530-nm excitation filter, 590-nm emission filter, and 570-nm dichroic mirror). The temperature was adjusted to 25°C, and the results were read at 15-s intervals for 0.5 h. Assays were repeated three times in triplicate; one representative data set is reported. The mean RFU for each sample were calculated and plotted using Prism 9. Experiments were reproduced three times, and one representative data set is reported.

### PG isolation.

Biological replicates were grown to mid-logarithmic or stationary phase in 400 mL of Luria broth. Cells were centrifuged (Avanti JXN-26 Beckman Coulter centrifuge and Beckman Coulter JA-10 rotor) at 7,000 × *g* for 0.5 h at 4°C, resuspended in chilled 6 mL PBS, and lysed via dropwise addition to boiling 8% sodium dodecyl sulfate (SDS). PG was further purified as previously described (84). Briefly, muropeptides were cleaved from PG by Cellosyl muramidase (Hoechst, Frankfurt am Main, Germany), reduced with sodium borohydride, and separated on a 250- by 4.6-mm 3-μm Prontosil 120-3-C_18_ AQ reversed-phase column (Bischoff, Leonberg, Germany). The eluted muropeptides were detected by the absorbance at 205 nm. Eluted peaks were designated based on known published chromatograms (20, 42, 57); new peaks were analyzed by MS/MS, as previously described (20).

### Activity assays.

PBP7 activity assays were carried out in a final volume of 50 μL containing 20 mM HEPES (pH 5.0, 6.0, or 7.5), 50 mM NaCl, and 2 μM PBP7 or PBP7_S131A_. PG from E. coli D456 was added, and the reaction mixture was incubated at 37°C for 16 h. The reaction was stopped by boiling the samples for 10 min. The reaction mixture was reduced with sodium borohydride and acidified to pH 4.0 to 4.5. E. coli D456 PG under pH 5.0 buffer conditions served as a control. Muropeptides were analyzed as previously described (84).

ElsL activity assays were carried out in a final volume of 50 μL containing 20 mM NaP (pH 5.0) and 10 μM ElsL or ElsL_C138S_. PG from E. coli BW25113 (WT) (tetra-muropeptide rich) or CS703-1 (multiple mutations in penicillin-binding proteins) (penta-muropeptide rich) was added, and the reaction mixture was incubated at 37°C for 4 h. The reaction was stopped by boiling the samples for 10 min. Muropeptides were reduced with sodium borohydride. Muropeptides were analyzed as previously described (84).

### Cell fractionation.

Cells were grown to mid-logarithmic or stationary phase and normalized to an OD_600_ of 0.75 in 20 mL. Cultures were washed twice with chilled 1× PBS plus 0.1% gelatin (PBSG), resuspended in 2 mL chilled PBSG containing 2 mg/mL polymyxin B sulfate (MilliporeSigma), and then agitated for 0.5 h at 4°C. Spheroplasts were pelleted at 20,000 × *g* for 0.5 h at 4°C. The remaining supernatant was centrifuged at 20,000 × *g* for 0.5 h at 4°C. The supernatant was collected and saved as the periplasmic fraction. Previously pelleted spheroplasts were resuspended in 1 mL 10 mM HEPES buffer solution (Gibco), and 100 μL was collected as whole spheroplasts. The remaining spheroplasts were sonicated for 15 s on and 15 s off 10 times at 60% amplitude (Qsonica Q125 sonicator). Lysed spheroplasts were pelleted at 16,000 × *g* for 0.5 h at 4°C. The supernatant was centrifuged for another 0.5 h at 16,000 × *g* at 4°C. The insoluble pellet was saved as the total membrane fraction. The soluble supernatant was saved as the cytoplasmic fraction. Experiments were reproduced three times, and one representative data set is reported.

### Immunoblot assays.

ElsL, OmpA, and RpoA polyclonal antibodies were generated (Thermo Fisher Scientific) from rabbit immunization with peptide fragments. Briefly, a 20-amino-acid peptide from ElsL (ELFDLVSEDALVYLSEQSLT), a 19-amino-acid peptide from OmpA (KEGRAMNRRVFATITGSR), and a 19-amino-acid peptide from RpoA (ENWPPASLRMDDRFAYRSR) predicted to be solvent exposed were selected from the primary sequence of A. baumannii ATCC 17978, synthesized, and used to generate each specific antibody from rabbits. The collected serum was tested for reactivity in an enzyme-linked immunosorbent assay (ELISA) with peptide fragments and via Western blotting against whole-cell lysates.

All Western blot analyses were performed using 4 to 12% Bis-Tris 10-well protein gels (Invitrogen) and NuPage morpholineethanesulfonic acid (MES) SDS running buffer (Novex). Gels were transferred with NuPage transfer buffer (Novex) to 0.45-μm polyvinylidene difluoride (PVDF) membranes (Amersham Hybond). All blots were blocked in 5% milk and 1× Tris-buffered saline (TBS) for 2 h. For primary rabbit antisera, anti-ElsL and anti-RpoA were used at a 1:750 dilution, while anti-OmpA was used at 1:1,000. Anti-rabbit horseradish peroxidase (HRP) secondary antibody was used at 1:10,000 (Thermo Fisher Scientific). The penta-his primary anti-mouse antibody was used at 1:500 (Invitrogen). Anti-mouse HRP secondary antibody was used at 1:10,000 (Invitrogen). SuperSignal West Pico Plus (Thermo Fisher Scientific) was applied to detect relative protein concentrations.

For localization assays, whole-cell lysates, whole spheroplasts, and membrane fractions were mixed with 1× loading dye containing 4% 2-mercaptoethanol (Fisher Chemical) and boiled for 10 min. Ten microliters of each sample was used. One hundred thirty-two microliters of the periplasmic or cytoplasmic fraction was added to 66 μL of 3× loading buffer containing 4% 2-mercaptoethanol (Fisher Chemical) and boiled for 10 min. Sixty microliters of each sample was used. Each sample was loaded into 4 to 12% Bis-Tris 10-well protein gels (Invitrogen) for immunoblotting.

For protein purification, 1 μg of purified protein was combined with 3× loading buffer containing 4% 2-mercaptoethanol and boiled for 10 min. The sample was loaded into a 4 to 12% Bis-Tris 10-well protein gel (Invitrogen) for immunoblotting.

### MIC determination.

MICs were determined using the broth microdilution (BMD) method, as previously outlined (85). Cultures grown overnight were back-diluted to an OD_600_ of 0.01, and 100 μL of cells was added to each well of a 96-well round-bottom polypropylene plate (Grenier Bio-One). Meropenem diluted in water was serially diluted, and 150 μL of each meropenem serial dilution was also added to each well. Plates were incubated overnight at 37°C, and growth was measured by reading the OD_600_ after 24 h of incubation. The lowest concentration of meropenem at which no bacterial growth was observed was determined to be the MIC. Assays were repeated three times in triplicate; one representative data set is reported.

### Statistical analysis.

Tests for significance in cell morphology, fluorescence intensity, and outer membrane vesicle production were conducted using Student’s *t* test (two-tailed distribution with two-sample, equal-variance calculations). Statistically significant differences between relevant strains possessed *P* values of <0.05.

### Data availability.

The sequencing data have been deposited in the National Center for Biotechnology Information’s Gene Expression Omnibus (accession number GSE190441).
