# Supplementary material for: Peptidoglycan Recycling Promotes Outer Membrane Integrity and Carbapenem Tolerance in Acinetobacter baumannii
Source: mBio. 2022 May 31;13(3):e01001-22. doi: 10.1128/mbio.01001-22 (PMC9239154; doi:10.1128/mbio.01001-22)
Supplement: TABLE S1 [file mbio.01001-22-s0001.docx]

| **Table S1: Muropeptide composition of wild type and Δ*pbpG A. baumannii* strain ATCC 17978.** | | | | | | | | | | |  |
| --- | --- | --- | --- | --- | --- | --- | --- | --- | --- | --- | --- |
|  |  |  | | | | | | | | |  |
|  |  | **Relative % of each muropeptide^a^** | | | | | | | | |  |
| **Peak No.** | **Muropeptide** | **WT Logarithmic** | **WT Stationary** | | **Δ*pbpG* Logarithmic** | | | | **Δ*pbpG* Stationary** | |  |
| **1** | **Tri** | 3.0 ± 0.4 | 3.2 ± 0.0 | | 1.5 ± 0.2 | | | | 2.0 ± 0.1 | |  |
| **2** | **Tri-D-Asn** | 0.9 ± 0.1 | 0.1 ± 0.2 | | 0.1 ± 0.3 | | | | 0.1 ± 0.2 | |  |
| **3** | **Tri-D-Lys** | 2.2 ± 0.7 | 4.8 ± 0.0 | | 1.0 ± 0.0 | | | | 1.1 ± 0.2 | |  |
| **4** | **TetraGly4** | 0.0 ± 0.0 | 0.7 ± 0.1 | | 0.0 ± 0.0 | | | | 0.0 ± 0.0 | |  |
| **5** | **Tetra-D-Lys** | 0.0 ± 0.0 | 2.6 ± 0.0 | | 0.0 ± 0.0 | | | | 0.0 ± 0.0 | |  |
| **6** | **Tetra** | 17.6 ± 0.5 | 17.5 ± 0.4 | | 16.1 ± 0.2 | | | | 14.6 ± 0.3 | |  |
| **7** | **Tetra-D-Arg** | 0.0 ± 0.0 | 0.4 ± 0.0 | | 0.0 ± 0.0 | | | | 0.1 ± 0.2 | |  |
| **7B** | **Penta** | 0.0 ± 0.0 | 0.5 ± 0.0 | | 1.3 ± 0.0 | | | | 0.4 ± 0.0 | |  |
| **8** | **TetraTriDapGly4** | 0.3 ± 0.0 | 0.8 ± 0.0 | | 0.0 ± 0.0 | | | | 0.2 ± 0.0 | |  |
| **9** | **TriTri(Dap)/TriTriDap-D-Lys** | 0.5 ± 0.1 | 0.5 ± 0.0 | | 0.1 ± 0.2 | | | | 0.0 ± 0.0 | |  |
| **10** | **TetraTri(Dap)** | 0.0 ± 0.0 | 1.8 ± 0.1 | | 0.0 ± 0.0 | | | | 0.4 ± 0.0 | |  |
| **11** | **TetraTri** | 4.3 ± 0.2 | 3.4 ± 0.1 | | 3.1 ± 0.0 | | | | 1.9 ± 0.0 | |  |
| **12** | **TetraTri-D-Lys** | 0.6 ± 0.0 | 3.7 ± 0.0 | | 0.5 ± 0.0 | | | | 1.0 ± 0.0 | |  |
| **13** | **TetraTri-D-Lys** | 0.4 ± 0.0 | 0.7 ± 0.0 | | 0.4 ± 0.0 | | | | 0.0 ± 0.0 | |  |
| **14** | **TetraTri-D-Arg** | 0.5 ± 0.1 | 6.0 ± 0.0 | | 2.0 ± 0.0 | | | | 1.7 ± 0.0 | |  |
| **15** | **TetraTetra** | 36.2 ± 0.9 | 22.9 ± 0.4 | | 39.6 ± 0.6 | | | | 35.7 ± 0.2 | |  |
| **15B** | **TetraPenta** | 0.5 ± 0.1 | 1.0 ± 0.1 | | 2.9 ± 0.0 | | | | 1.0 ± 0.1 | |  |
| **16** | **TetraTetraTri or TetraTetraTriDap** | 0.1 ± 0.0 | 0.9 ± 0.0 | | 0.9 ± 0.0 | | | | 1.2 ± 0.1 | |  |
| **17** | **TetraTetraTri or TetraTetraTriDap** | 0.3 ± 0.0 | 1.6 ± 0.0 | | 0.3 ± 0.2 | | | | 0.4 ± 0.0 | |  |
| **18** | **TriTriDap-D-Met** | 0.6 ± 0.0 | 0.5 ± 0.0 | | 0.2 ± 0.4 | | | | 0.6 ± 0.0 | |  |
| **19** | **TetraTetraTetra** | 17.9 ± 0.0 | 11.8 ± 0.3 | | 16.9 ± 0.2 | | | | 18.6 ± 0.1 | |  |
| **20** | **TetraTri-D-Met** | 0.1 ± 0.2 | 0.7 ± 0.0 | | 0.7 ± 0.0 | | | | 0.2 ± 0.0 | |  |
| **21** | **TetraTriAnh / TetraTetraTetraTri** | 4.4 ± 0.1 | 2.4 ± 0.2 | | 3.9 ± 0.0 | | | | 4.7 ± 0.0 | |  |
| **22** | **TetraTetraAnh I** | 1.4 ± 0.0 | 0.8 ± 0.0 | | 2.0 ± 0.0 | | | | 2.2 ± 0.1 | |  |
| **23** | **TetraTetraAnh II** | 0.7 ± 0.1 | 0.9 ± 0.0 | | 1.0 ± 0.0 | | | | 1.4 ± 0.1 | |  |
| **24** | **TetraTetraTetraAnh** | 2.5 ± 0.1 | 1.6 ± 0.0 | | 2.2 ± 0.0 | | | | 3.2 ± 0.0 | |  |
| **Sum of known peaks** | | 95.8 ± 0.2 | 91.7 ± 0.1 | | 95.8 ± 0.2 | | | | 92.8 ± 0.4 | |  |
|  |  |  |  | |  | | | |  | |  |
| **Monomers (Total)** | | 24.8 ± 0.6 | 32.3 ± 0.3 | | 20.9 ± 0.0 | | | | 19.7 ± 0.0 | |  |
| **Monomers with modification** | | 3.2 ± 0.6 | 9.3 ± 0.2 | | 1.1 ± 0.3 | | | | 1.4 ± 0.3 | |  |
| **Monomer tri** | | 3.2 ± 0.5 | 3.5 ± 0.0 | | 1.6 ± 0.2 | | | | 2.2 ± 0.1 | |  |
| **Monomer tri-D-Asn** | | 0.9 ± 0.1 | 0.1 ± 0.3 | | 0.1 ± 0.3 | | | | 0.1 ± 0.2 | |  |
| **Monomer tri-D-Lys** | | 2.3 ± 0.7 | 5.2 ± 0.0 | | 1.0 ± 0.0 | | | | 1.1 ± 0.2 | |  |
| **Monomer tetraGly4** | | 0.0 ± 0.0 | 0.8 ± 0.1 | | 0.0 ± 0.0 | | | | 0.0 ± 0.0 | |  |
| **Monomer tetra-D-Lys** | | 0.0 ± 0.5 | 2.8 ± 0.0 | | 0.0 ± 0.0 | | | | 0.0 ± 0.0 | |  |
| **Monomer tetra** | | 18.4 ± 0.5 | 19.0 ± 0.4 | | 16.8 ± 0.2 | | | | 15.7 ± 0.4 | |  |
| **Monomer tetra-D-Arg** | | 0.0 ± 0.0 | 0.4 ± 0.0 | | 0.0 ± 0.0 | | | | 0.1 ± 0.2 | |  |
| **Monomer penta** | | 0.0 ± 0.0 | 0.5 ± 0.0 | | 1.4 ± 0.0 | | | | 0.4 ± 0.0 | |  |
|  |  |  |  | |  | | | |  | |  |
| **Dimers (Total)** | | 52.5 ± 0.4 | 50.3 ± 0.2 | | 58.8 ± 0.2 | | | | 55.1 ± 0.2 | |  |
| **Dimers with modification** | | 2.9 ± 0.5 | 14.1 ± 0.1 | | 4.1 ± 0.7 | | | | 4.0 ± 0.1 | |  |
| **Dimer chain ends (anhydroMur*N*Ac)** | | 6.8 ± 0.0 | 4.4 ± 0.2 | | 7.2 ± 0.0 | | | | 9.0 ± 0.1 | |  |
|  |  |  |  | |  | | | |  | |  |
| **Trimers (Total)** | | 22.6 ± 0.2 | 17.4 ± 0.5 | | 21.2 ± 0.2 | | | | 25.2 ± 0.1 | |  |
| **Trimer chain ends (anhydroMur*N*Ac)** | | 2.6 ± 0.1 | 1.7 ± 0.0 | | 2.3 ± 0.1 | | | | 3.5 ± 0.0 | |  |
|  |  |  |  | |  | | | |  | |  |
| **Tripeptides (Total)** | | 13.0 ± 1.2 | 20.8 ± 0.4 | | 9.0 ± 0.7 | | | | 10.1 ± 0.2 | |  |
| **Tripeptides with modifications** | | 5.2 ± 0.9 | 13.0 ± 0.3 | | 3.4 ± 1.0 | | | | 3.6 ± 0.1 | |  |
| **Tetrapeptides (Total)** | | 86.3 ± 1.3 | 77.5 ± 0.4 | | 89.0 ± 0.9 | | | | 88.9 ± 0.1 | |  |
| **Tetrapeptides with modifications** | | 0.9 ± 0.2 | 10.5 ± 0.1 | | 1.9 ± 0.0 | | | | 1.8 ± 0.3 | |  |
| **Pentapeptides** | | 0.2 ± 0.1 | 1.0 ± 0.0 | | 2.9 ± 0.0 | | | | 1.0 ± 0.1 | |  |
|  |  |  |  | |  | | | |  | |  |
| **3-3 Crosslinks** | | 1.1 ± 0.1 | 2.9 ± 0.1 | | 0.6 ± 0.3 | | | | 1.2 ± 0.1 | |  |
| **Chain ends (anhydroMur*N*Ac)** | | 4.3 ± 0.0 | 2.8 ± 0.1 | | 4.3 ± 0.0 | | | | 5.7 ± 0.1 | |  |
|  |  |  |  | |  |  | | | |  | |
| **Degree of crosslinkage** | | 41.4 ± 0.3 | 36.7 ± 0.2 | | 43.6 ± 0.0 | | | | 44.3 ± 0.0 | |  |
| **% peptides in cross-links** | | 75.2 ± 0.6 | 67.7 ± 0.3 | | 79.1 ± 0.0 | | | | 80.3 ± 0.1 | |  |
| **^a^Values are mean ± variation of two biological repeats.** | | |  |  | | |  |  |  |  |  |
