# Supplementary material for: Peptidoglycan Recycling Promotes Outer Membrane Integrity and Carbapenem Tolerance in Acinetobacter baumannii
Source: mBio. 2022 May 31;13(3):e01001-22. doi: 10.1128/mbio.01001-22 (PMC9239154; doi:10.1128/mbio.01001-22)
Supplement: TABLE S2 [file mbio.01001-22-s0002.docx]

| **Table S2:** Strains and plasmids used in this study. | | |
| --- | --- | --- |
| **Strain/Plasmid** | **Description** | **Reference/Source** |
| **Strains** |  |  |
| *E. coli* C2987 | chemically competent wild type, K-12 | New England Biolabs |
| *E. coli* C2527 | chemically competent BL-21 | New England Biolabs |
| *A. baumannii* ATCC 17978 | wild type | ATCC (1) |
| *A. baumannii* ATCC 19606 | wild type | ATCC (2) |
| *A. baumannii* AYE | wild type | ATCC (3) |
| *A. baumannii* AR Bank #273 | Clinical isolate | (4) |
| *A. baumannii* AR Bank #280 | Clinical isolate | (4) |
| *A. baumannii* AR Bank #300 | Clinical isolate | (4) |
| *A. baumannii* ATCC 17978 | Δ*ompA* | This Study |
| *A. baumannii* ATCC 17978 | Δ*ompA* / pOmpA | This Study |
| *A. baumannii* ATCC 17978 | Δ*lpxM* | (5) |
| *A. baumannii* ATCC 17978 | Δ*lpxM* / pLpxM | (5) |
| *A. baumannii* ATCC 17978 | Δ*pbpG* | This Study |
| *A. baumannii* ATCC 17978 | Δ*pbpG* / pPBP7 | This Study |
| *A. baumannii* ATCC 17978 | Δ*elsL* | (6) |
| *A. baumannii* ATCC 17978 | Δ*elsL* / pElsL | (6) |
| *A. baumannii* ATCC 17978 | Δ*ampD* | This Study |
| *A. baumannii* ATCC 17978 | Δ*ampD* / pAmpD | This Study |
| *A. baumannii* ATCC 17978 | Δ*adeIJK* | This Study |
| *A. baumannii* ATCC 17978 | Δ*ompW* | This Study |
| *A. baumannii* ATCC 17978 | Δ*A1S_3492* | This Study |
| **Plasmids** |  |  |
| pMMB67EH | Amp^R^ | (7) |
| pABBR | Amp^R^ | (8) |
| pABBRKn | pABBR_MCS with the *Kan^R^* gene from pKD4 inserted into the PvuI site, Kn^R^ | (9) |
| pMMB67EHKn | pMMB67EH with the *Kan^R^* gene from pKD4 inserted into the PvuI site, Kn^R^ | (6) |
| pJNW684 | Tn vector, Amp^R^, Kan^R^ | (10) |
| pAT03 | pMMB67EH with FLP recombinase, Amp^R^ | (8) |
| pAT04 | pMMB67EH with REC_Ab_ system, Tet^R^ | (8) |
| pKD4 | Kan^R^ | (11) |
| pT7-7 | Amp^R^ | (12) |
| pT7-7Kn | pT7-7 with the *Kan^R^* gene from pKD4 inserted into the PvuI site, Kn^R^ | This study |
| pUC19 | Amp^R^ | (13) |
| pElsL | pMMB67EHKn with the *elsL* (A1S_2806) gene and IPTG inducible promoter inserted into the KpnI and SalI sites, Kn^R^ | (6) |
| pUC19::ElsL_C138S_ | pUC19 with *elsL*_C138S_ (A1S_2806) gene cloned into the BamHI sites, Amp^R^ | (6) |
| pElsL_C138S_ | pMMB67EHKn with the *elsL_C138S_* (A1S_2806) gene and IPTG inducible promoter inserted into the KpnI and SalI sites, Kn^R^ | (6) |
| pElsL-His_8_-tag | pT7-7 with *elsL*_C138S_ (A1S_2806) cloned into the NdeI and BamHI sites, Kn^R^ | This study |
| pElsL_C138S_-His_8_-tag | pT7-7 with *elsL*_C138S_ (A1S_2806) cloned into the NdeI and BamHI sites, Kn^R^ | This study |
| pPBP7 | pMMB67EHKn with the *pbpG* (A1S_0237) gene and native promoter inserted into the XhoI and KpnI sites, Kn^R^ | This study |
| pUC19::PBP7_S131A_ | pUC19 with *pbpG* _S131A_ (A1S_0237) gene cloned into the BamHI sites, Amp^R^ | This study |
| pPBP7-His_8_-tag | pT7-7 with *pbpG* (A1S_0237) cloned into the NdeI and BamHI sites, Kn^R^ | This study |
| pPBP7_S131A_-His_8_-tag | pT7-7 with *pbpG* _S131A_ (A1S_0237) cloned into the NdeI and BamHI sites, Kn^R^ | This study |
| pOmpA | pMMB67EH with *ompA* (A1S_2840) cloned into the KpnI and SalI sites, Amp^R^ | This study |
| pLpxM | pMMB67EH with *lpxM* (A1S_2609) cloned into the KpnI and SalI sites, Amp^R^ | (5) |
|  |  |  |

**References:**

1. Baumann P, Doudoroff M, Stanier RY. 1968. A study of the *Moraxella* group. II. Oxidative-negative species (genus *Acinetobacter*). *J Bacteriol* 95:1520–1541.

2. Bouvet PJM, Grimont PAD. 1986. Taxonomy of the Genus *Acinetobacter* with the Recognition of *Acinetobacter baumannii sp.* nov., *Acinetobacter haemolyticus sp.* nov., *Acinetobacter johnsonii sp.* nov., and *Acinetobacter junii sp.* nov. and Emended Descriptions of *Acinetobacter calcoaceticus* and *Acinetobacter lwoffii*. *International Journal of Systematic and Evolutionary Microbiology* 36:228–240.

3. Fournier P-E, Vallenet D, Barbe V, Audic S, Ogata H, Poirel L, Richet H, Robert C, Mangenot S, Abergel C, Nordmann P, Weissenbach J, Raoult D, Claverie J-M. 2006. Comparative genomics of multidrug resistance in *Acinetobacter baumannii. PLoS Genet* 2:e7.

4. CDC. 2021. CDC & FDA antibiotic resistance isolate bank. Atlanta (GA).

5. Boll JM, Tucker AT, Klein DR, Beltran AM, Brodbelt JS, Davies BW, Trent MS. 2015. Reinforcing Lipid A Acylation on the Cell Surface of *Acinetobacter baumannii* Promotes Cationic Antimicrobial Peptide Resistance and Desiccation Survival. *mBio* 6:e00478-00415.

6. Kang KN, Kazi MI, Biboy J, Gray J, Bovermann H, Ausman J, Boutte CC, Vollmer W, Boll JM. 2021. Septal Class A Penicillin-Binding Protein Activity and ld-Transpeptidases Mediate Selection of Colistin-Resistant Lipooligosaccharide-Deficient *Acinetobacter baumannii. mBio* 12:e02185-20.

7. Rozen S, Skaletsky H. 1999. Primer3 on the WWW for General Users and for Biologist Programmers, p. 365–386. *In* Bioinformatics Methods and Protocols. Humana Press, New Jersey.

8. Tucker AT, Nowicki EM, Boll JM, Knauf GA, Burdis NC, Trent MS, Davies BW. 2014. Defining Gene-Phenotype Relationships in *Acinetobacter baumannii* through One-Step Chromosomal Gene Inactivation. *mBio* 5.

9. Boll JM, Crofts AA, Peters K, Cattoir V, Vollmer W, Davies BW, Trent MS. 2016. A penicillin-binding protein inhibits selection of colistin-resistant, lipooligosaccharide-deficient *Acinetobacter baumannii*. *Proc Natl Acad Sci USA* 113:E6228–E6237.

10. Wang N, Ozer EA, Mandel MJ, Hauser AR. 2014. Genome-Wide Identification of Acinetobacter baumannii Genes Necessary for Persistence in the Lung. *mBio* 5:e01163-14.

11. Datsenko KA, Wanner BL. 2000. One-step inactivation of chromosomal genes in *Escherichia coli* K-12 using PCR products. *Proc Natl Acad Sci USA* 97:6640–6645.

12. William Studier F, Rosenberg AH, Dunn JJ, Dubendorff JW. 1990. [6] Use of T7 RNA polymerase to direct expression of cloned genes, p. 60–89. *In* Methods in Enzymology. Elsevier.

13. Yanisch-Perron C, Vieira J, Messing J. 1985. Improved M13 phage cloning vectors and host strains: nucleotide sequences of the M13mpl8 and pUC19 vectors. Gene 33:103–119.
