# Supplementary material for: Peptidoglycan Recycling Promotes Outer Membrane Integrity and Carbapenem Tolerance in Acinetobacter baumannii
Source: mBio. 2022 May 31;13(3):e01001-22. doi: 10.1128/mbio.01001-22 (PMC9239154; doi:10.1128/mbio.01001-22)
Supplement: TABLE S3 [file mbio.01001-22-s0003.docx]

| **Table S3:** Oligonucleotides used in this study | |
| --- | --- |
| **Oligo Name** | **Sequence (5’ to 3’)** |
| **Deletion Primers** |  |
| 17978 *ompA* Kan-FRT 5’ | TCAAGTGTTTGTATGATTCAAATGTGAATAGCTTAAAAATAATACTGGGGTAAAAAAATATCTCAGGGGCCAATAAATTTAGGCTGAGCTTGAACAACAATTGTTATCTCTGGAGGATATCCATGagcgattgtgtaggctggagctgcttcg |
| 17978 *ompA* Kan-FRT 3’ | ATTTTAAGTAATGATTGGAAGAGATTATGAATCAGGAGATTTACAAATGACCAAATATTTTAAAAATCGCCATAAAAAAAGCGACTCTAACGAGTCGCTTTTTTACTGTTCAAGAACTCAAATTAatatcctccttagttcctattccg |
| 17978 *ompA*-kn verify 5’ | ACTGGTAAAATCACGGCAAGCG |
| 17978 *ompA*-kn verify 3’ | CCTCGTGCTCAACATCGTAGAAATAG |
| 17978 *ampD* Kan-FRT 5’ | ATTAACTGTTTTAAACTAAAACAATTGGCAAGCAATTTATTGGCATCTCAATGAAGCAAATCACACCGTATGAAGTTATAGATGGACAATTAAAAGGGGCGAGACAAGTACCTTCTCCAAATTTTagcgattgtgtaggctggagctgcttcg |
| 17978 *ampD* Kan-FRT 3’ | TGCCATTTAAAATAAGGTCCCGGATCGGTTTTTCGGCCCGGTGCAATGTCTGAATGCCCTGCAATATGGTTTTTAATTTCAGGATAGGCCTGACGAATAGCAGTAACAACGTCAGTTAGCACTTCatatcctccttagttcctattccg |
| 17978 *ampD*-kan verify 5 | GACTGAGGTATGCTCATACGG |
| 17978 *ampD*-kan verify 3 | CGAAATCTTTACCCGCACCAAA |
| 17978 *pbpG*-Kn FRT F | AAAGCTTTATACCTTATATCTCAAATGTAAGGCATAATGATAGTAAGCGCAAATGTGTGTCACCCTGAGTCGAGTATTGTGCCGTGAAAAATTCTAAAAAGTCTTTAATGCATGTGCTAAGCATGatatcctccttagttcctattccg |
| 17978 *pbpG* -Kn FRT R | TATCTAATATGTAAAATCTGAGTTTTTATAAAAAGCGGCTGTTTAATACAGCCGTTTTTTTATGCTTTTTAAATGGCATAAAAAAACGCTTCTTAAAAGAAGCGTTTTTAAAAATAATTAAATTAagcgattgtgtaggctggagctgcttcg |
| 17978*pbpG* confirm Up | GCTTGCAATGGAATGACAAAATTAGCAATC |
| 17978*pbpG* confirm Dwn | GATACAGCAATTAAACAATGTGCTGATGCAG |
| 17978 *adeIJK*-Kan 5’ | TCGCTGGTTGGGGCATTGGTTTTATTTGGATTGCACTGCTTTGGCTCTGGTTATTACAAACACAAAGTAGGTTAAGTAGAAAACAAATATATTTTTAGATTTTATCTAAACGAGGTGGAACAATGagcgattgtgtaggctggagctgcttcg |
| 17978 *adeIJK*-Kan 3’ | TACTTAATAACATTGTTTTTTATCAATCTAAATTTCTTTAGCTTTCAAGATAAAAAATATTCAGATGAGTTGCAATAAAAAAGCCCACCGAAGTGAGCTTTTATAAGAAAGTGATTTAAAACTTAatatcctccttagttcctattccg |
| 17978 *adeIJK-kan* verify 5’ | GTTTATGCATCAACACTGGCTTG |
| 17978 *adeIJK-kan* verify 3’ | CTAATAATTCGGTATAACCCGT |
| 17978 *A1S_3492-kan* 5’ | ggaaaaaataagccatttttacaaaaagtctattaaagcactcttaatttattacttaatgggggaaaaccctccatgaaagcatactattctaggcgcaactgagtagaggagtagctttaatgagcgattgtgtaggctggagctgcttcg |
| 17978 *A1S_3492-kan* 3’ | tagatattttgtaaagccaatttatgtgcagtgatgagttaaatttacatcttttaaaagcagataaaaaagaagctcttaatgaaataagagcttcttttttaatatagttagaggtagttttaatatcctccttagttcctattccg |
| 17978 *A1S_3492-kan* verify 5’ | gccaagctttaaactcagaacaattctaagc |
| 17978 *A1S_3492-kan* verify 3’ | ctcactcctttgggctttaatcaaagcat |
| 17978 *ompW-kan* 5’ | atttcatgtcattttgaatgacgtcatgactgcccaccccttattgaatttatcaacttttagaagttatggcagctttttgtctgacttttagggcataaattacctgtagcggagacaacatg agcgattgtgtaggctggagctgcttcg |
| 17978 *ompW-kan* 3’ | gttcaaaaaagtattagagcaaaccgcttaaactgaaaaagaattcatataaaaagcttacccgagaggtaagctttttatttatacaaacaatgaatttatcaacttttagatgataaaaacta atatcctccttagttcctattccg |
| 17978 *ompW-kan* verify 5’ | gcgcgcatcttaaatgttgagtattttttaacc |
| 17978 *ompW-kan* verify 3’ | cctagagtttgatatggcagctgcta |
|  |  |
| **Complementation Primers** |  |
| 17978 o*mpA* CDS Kpnl | CGCGGTACCATGAAATTGAGTCGTATTGCACTTGCTAC |
| 17978 *ompA* CDS SalI | CGCGTCGACTTATTGAGCTGCTGCAGGAGCTGCC |
| 17978 *ampD* CDS BamHI | CGCGGATCCATGAAGCAAATCACACCGTATGAAGTTATAGAT |
| 17978 *ampD* CDS SalI | CGCGTCGACTCAAGTTTTTTTCTGTGCTAACAACTGCCTAAA |
| pABBR-*pbpG*-F XhoI | AAATTACTCGAGCTATTCTTCTATAGTGAGCGAATAGTTG |
| pABBR-*pbpG*-R KpnI | GTTGTCGGTACCTGCAACAATGGACCAAGTAAAAGATTCG |
| **Mutagenesis Primers** |  |
| *pbpG*_S131A_ Fwd | CAGGTGAGGTACTTTATAGTAAAAATACCAACGCATCAGTGCCGATCGCTGCAATTACCAAATTGATGACGGCAGTTGTAACGGCAGATGCCCGTTTAAACAT |
| *pbpG*_S131A_ Rev | ATGTTTAAACGGGCATCTGCCGTTACAACTGCCGTCATCAATTTGGTAATTGCAGCGATCGGCACTGATGCGTTGGTATTTTTACTATAAAGTACCTCACCTG |
| pUC19-*pbpG* BamHI 5' | CGCGGATCCATGTCTATTTTGCTTAGT |
| pUC19-*pbpG* BamHI 3' | CGCGGATCCTTAAATACGTTTTGGCAAAT |
| **Overexpression Primers** |  |
| pT7-*pbpG* NdeI | CGCCATATGTCTATTTTGCTTAGT |
| pT7-*pbpG* BamHI 8X-his | CGCGGATCCTTAATGGTGATGGTGATGGTGATGGTGAATACGTTTTGGCAAAT |
| **Sequencing Primers** |  |
| pMMB67EH seq fwd | CGGTTCTGGCAAATATTCTGAAA |
| pMMB67EH Seq3 | CTGCGTTCTGATTTAATCTGTAT |
| pABBR confirm 1 | GGGCTGACCGCTTCCT |
| pABBR confirm 2 | CGCTAGCAGCACGCCATAG |
